# Reversal of reserpine-induced depression and cognitive disorder in zebrafish by sertraline and Traditional Chinese Medicine (TCM)

**DOI:** 10.1186/s12993-018-0145-8

**Published:** 2018-06-14

**Authors:** Shuhui Zhang, Xiaodong Liu, Mingzhu Sun, Qiuping Zhang, Teng Li, Xiang Li, Jia Xu, Xin Zhao, Dongyan Chen, Xizeng Feng

**Affiliations:** 1 State Key Laboratory of Medicinal Chemical Biology, The Key Laboratory of Bioactive Materials, Ministry of Education, College of Life Science, Nankai University, Tianjin, 300071 China; 2 Tianjin Key Laboratory of Tumor Microenvironment and Neurovascular Regulation, Department of Histology and Embryology, School of Medicine, Nankai University, Tianjin, 300071 China; 3 The Institute of Robotics and Automatic Information Systems, Nankai University, Tianjin, 300071 China

**Keywords:** Depression behaviour, TCM, Colour preference, Monoamines, Zebrafish

## Abstract

**Background:**

With increased social pressure, individuals face a high risk of depression. Subsequently, depression affects cognitive behaviour and negatively impacts daily life. Fortunately, the Traditional Chinese Medicine Jia Wei Xiao Yao (JWXY) capsule is effective in reducing depression and improving cognitive behaviour.

**Methods:**

The constituents of JWXY capsule were identified by ultra-performance liquid chromatography and quadrupole time-of-flight mass spectrometry analyses. We analysed behaviours of depression-like zebrafish in the novel tank with an automatic 3D video-tracking system and conducted the colour preference test, as well detected physiological changes after sertraline and JWXY capsule treatments.

**Results:**

Both sertraline and JWXY capsule rescued the decreased locomotive behaviour and depression phenotype of zebrafish caused by reserpine. JWXY capsule especially improved the inhibited exploratory behaviour caused by reserpine. In addition, with the onset of depressive behaviour, zebrafish exhibited alterations in cognitive behaviour as indicated by colour preference changes. However, compared with sertraline, JWXY capsule was more efficaciously in rescuing this change in the colour preference pattern. Moreover, an increased level of cortisol, increased expression of tyrosine hydroxylase (TH) and decreased monoamine neurotransmitters, including serotonin (5-HT) and noradrenaline, were involved in the depressive behaviours. In addition, sertraline and JWXY capsule rescued the depressive phenotype and cognitive behaviour of zebrafish by altering the levels of endogenous cortisol and monoamine neurotransmitters.

**Conclusions:**

JWXY capsule was more effectively than sertraline in rescuing reserpine-induced depression and cognitive disorder in zebrafish. Potentially, our study can provide new insights into the clinical treatment of depression and the mechanism of action of JWXY capsule.

**Electronic supplementary material:**

The online version of this article (10.1186/s12993-018-0145-8) contains supplementary material, which is available to authorized users.

## Background

Major depressive disorder (MDD), one of the most common brain disorders, usually has a high rate of comorbidity with other psychiatric disorders [1]. Depressive disorder, characterised by decreased activity, a significant and lasting low mood, and slowed thinking and cognitive function [2, 3], markedly reduces quality of life. Psychiatric disorders such as psychosis, depression, and other mood disorders may have multigenic and multifactorial aetiologies [4]. Fortunately, improvements in the diagnosis and treatment of depression are increasing. Monoamines play a key role in the regulation of brain functions in animals and humans [5]. Monoamine neurotransmitters, including serotonin (5-HT), dopamine (DA) and noradrenaline (NA), are implicated in the regulation of a large number of processes, such as motor control, social behaviour, cognition, sleep, appetite, and anxiety in vertebrates [6–9]. In zebrafish, 5-HT and DA are the two most studied monoamines [10, 11]. Serotonin (5-hydroxytryptamine, 5-HT) serves as both a neurotransmitter and hormone; in higher vertebrates, 5-HT acts throughout the body, including the central nervous system (CNS), peripheral nervous system, cardiovascular system, and endocrine system; it also participates in sensory perception and many behaviours [12]. Serotonin is involved in many behavioural functions, including the organization of defence, and its putative pathological correlate, anxiety and stress disorders [13]. Anxiety-like behaviour positively correlates with 5-HT content in the novel tank test [14]. Stress levels can be measured by the whole-body cortisol concentration [15]. Some compounds cause Parkinson’s disease-like behaviour due to decreased dopamine levels and locomotor activity [16, 17]. Thus, the study of monoamine neurotransmitters in the brain is indispensable for the treatment of depression.

For depression, the most widely used therapy is antidepressants, including monoamine oxidase inhibitors (MAOIs), tricyclic antidepressants (TCAs), serotonin and norepinephrine reuptake inhibitors (SNRIs) and selective serotonin reuptake inhibitors (SSRIs) [18]. For example, sertraline is one a SSRI. Although these antidepressant drugs are effectively relieve depression, they have several concerning side effects, such as headache, agitation or sedation, vomiting, and fatigue [19, 20]. Therefore, identifying a better antidepressant is necessary; this need has led researchers to focus on natural medicine, including Traditional Chinese Medicine (TCM). TCM has a long history of prevention and treatment of depression dating as far back as 2000 years ago. When treating depression, TCM starts at the whole-body level, considering not only the psychological problems that result from a patient’s nervous system disorder but also the changes in the Zang-Fu organs, qi and blood [18]. TCM, such as Jia Wei Xiao Yao (JWXY) capsule, can provide a reliable clinical curative effect comparable to that of Western medicine. In addition, TCM is much more affordable and has fewer side effects. However, a lack of rigorous clinical research has counteracted the unique advantage of TCM and seriously impeded its worldwide popularization and application. JWXY capsule can soothe the liver and reduce heat, strengthen the spleen and nourish the blood. Based on experiences with TCM, JWXY capsule exerts various actions, including soothing the liver and improving the circulation of qi to relieve depression. In China, JWXY capsule has been commonly recognized as a safe and effective prescription in the treatment of depressive disorder [18, 21–23]. However, the effects and mechanism of action of JWXY capsule remain poorly understood.

In the literature, several assays have been reported to measure behavioural learning changes in adult zebrafish such as the rotating escape test, bite test, novel tank test, place preference test, T-maze, plus maze and Y-maze assays [24, 25]. Most of tools used to assess learning and memory in animal models involve visual stimuli, including colour preferences. Zebrafish can discriminate colours and display spontaneous approach or avoidance behaviours. Some studies support colour-based learning and memory paradigms or experiments involving aversion, anxiety or fear in zebrafish [26, 27]. Zebrafish show a preference for blue and green and avoided yellow and red [28]. The zebrafish visual system includes retinas with cones sensitive to red, green, blue, and ultraviolet; moreover zebrafish are diurnal animals, which makes them an ideal model for developing research on cognitive responses to visual signals [29, 30].

In cognitive research, the zebrafish has become increasingly popular and has advantages in behavioural brain research due to its elaborate brain structure, simplicity and neurochemistry, which offers translational relevance to humans [31–33]. In addition, the zebrafish is an ideal and promising model organism for pharmacology [4, 18, 34–36], disease [35, 37], embryology and development studies [38, 39] because it shares many genes, protein products and molecular pathways with mammals [40]. There are also studies on the relationship between emotion regulation and colour preference in zebrafish [41]. Zebrafish may become a translationally relevant study species for the analysis of the mechanisms of learning and memory changes associated with psychopharmacological treatment of anxiety/depression [42].

Compared with 2D approaches, a 3D approach improves data integrity by using two videos and may help reduce the number of experimental subjects. We used two cameras covering the dorsal and lateral view to record fish behaviour in a novel tank. A 3D approach integrates the position information from the top and front views, which is essential to measure depression-like behaviour in zebrafish [43]. 2D approaches have also played a pivotal role in elucidating the neurobehavioural underpinnings of fish behaviour [43]. Hence, we utilized a camera from the top view to record the preference of zebrafish for different colours after pharmacological manipulations.

Reserpine causes depression by depleting monoamines and is widely used to induce depression-like phenotypes by pharmacological manipulation in zebrafish [5]. Therefore, in this study, we performed comparative analysis of the curative effect of sertraline and JWXY capsule treatment for reserpine-induced depression-like behaviour in zebrafish by examining behaviour and the concentrations of three monoamine neurotransmitters and the hormone cortisol. Sertraline and JWXY capsule rescued depressive behaviour and colour preference, accompanied by changes in monoamines and cortisol. The purpose of our study was to evaluate the effects of sertraline and JWXY capsule on behaviour, cognitive ability and biochemical parameters in zebrafish with depression induced by reserpine.

## Methods

### Zebrafish

Zebrafish (AB strain) were maintained in a fish-farming system at the State Key Laboratory of Medicinal Chemical Biology, Nankai University. The room was maintained at a constant temperature of 28.5 °C on a constant light cycle (14 h light/10 h dark), and the water (KCl 0.05 g/L, NaHCO_3_ 0.025 g/L, NaCl 3.5 g/L, and CaCl_2_ 0.1 g/L) was circulated continuously. The zebrafish were fed freshly hatched brine shrimp twice daily. All of the experimental protocols and procedures involving zebrafish were approved by the Committee for Animal Experimentation of the College of Life Science at Nankai University (no. 2008) and were performed in accordance with the NIH Guide for the Care and Use of Laboratory Animals (no. 8023, revised in 1996).

### Behavioural test apparatuses and behavioural parameters

**Fig. 1:**
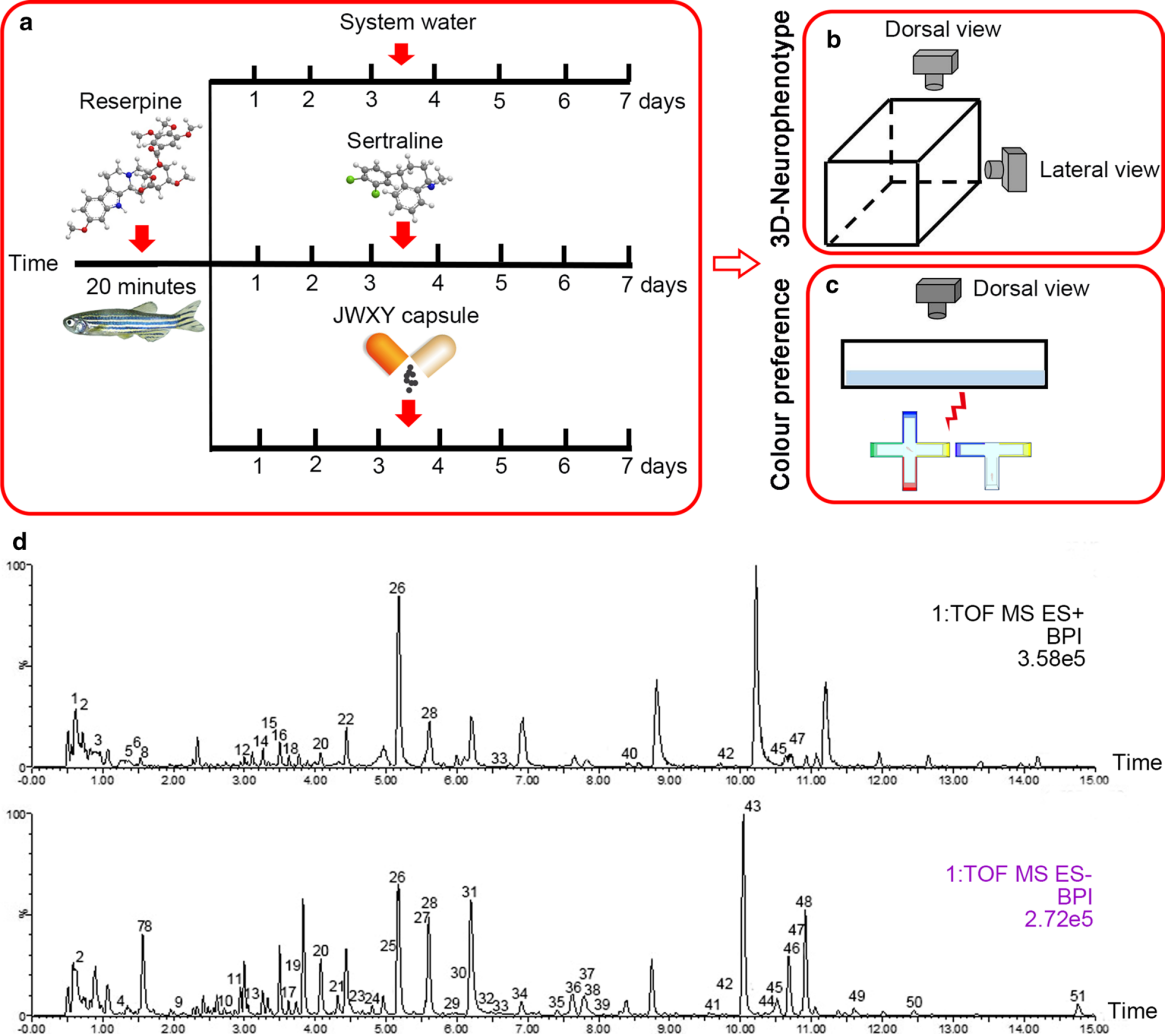
Experimental paradigm and ESI–MS spectra of JWXY capsule. **a** Timeline of the procedure for drug delivery and schematic diagram of the apparatus used for behavioural phenotyping in the novel tank (**b**) and colour preference behaviour (**c**). **d** ESI–MS spectra in the positive and negative ion voltage mode of JWXY capsule (1–15 min). Some of the constituents are labelled in the spectra

Behavioural apparatuses were designed according to previous studies [44, 45]. A novel tank, composed of transparent Plexiglass, was a 5 L rectangular box (23 cm length * 15 cm width * 15 cm depth) used to assess the depressive behaviour of zebrafish. We divided the tank into two equal horizontal portions virtually by marking a midline on the outside walls. The region above this midline indicated the “top” of the novel tank, while the area below indicated the “bottom” of the novel tank. The novel tank was placed over a light source, a light-emitting diode (LED) array, with an acrylic diffuser located above the tank. The light source was composed of white light (500 lux) arrays and a transparent platform. Two charge-coupled device (CCD) cameras (MV-VS078FM, Microvision, 10 frames/s) were placed to obtain the top (dorsal) view and side (lateral) view of the moving zebrafish (Fig. 1b). The offset cross maze and T-maze (Fig. 1c) were designed based on previous research and composed of transparent Plexiglass. Every arm of the offset cross maze is 20 cm * 8.8 cm. The sides of the four arms are covered with four different colours (blue, green, red and yellow) made from polypropylene. The centre Section (8.8 cm * 8.8 cm) of the cross maze is a starting place for the fish indicated by None. The two opposite arms of the maze are 20 cm * 8.8 cm each and are covered with two different colours (blue and yellow) of polypropylene on the sides. The last arm is 20 cm * 8.8 cm and uncoloured. The last arm and centre Section (8.8 cm * 8.8 cm) of the T-maze is a starting place for the fish indicated by No. The maze is 10 cm deep and filled with 6.5 cm system water. A CCD camera (MV-VS078FM, Microvision, 10 frames/s) was fixed above the maze to obtain the top view of the moving zebrafish (Fig. 1c). A daylight lamp (500 lux) or natural light served as the light source. All apparatuses rested on a level, stable surface and were placed in a relatively sound-proof room to minimize the effect of noise when behavioural tests were conducted. A big black cloak forming a space covered all the experimental apparatuses to eliminate environmental interference.

Briefly, the behavioural parameters were defined according to the literature and previous research [44, 45]. The definitions of behavioural parameters that described depression in the novel tank are shown in Table 1. The definitions of behavioural parameters that described colour preferences in the maze are provided in Table 2.

**Table 1:** The definitions of behavioural parameters in the novel tank

Behavioural parameters	Definition
Total distance travelled (m)	The total distance in the novel tank
Average velocity (cm/s)	The direction and magnitude of zebrafish speed in the novel tank
Turn angle (°)	The total turning angle of zebrafish in the novel tank
Angular velocity (°/s)	The direction and magnitude of zebrafish angular speed in the novel tank
Meandering (°/s)	The degree of turning vs. travel distance
Average entry duration in the top (s)	The amount of time spent at the top of the novel tank during each crossing
Distance travelled in the top (m)	The total distance moved in the defined top part in the novel tank
Time spent in the top (s)	The total time spent in the top part of the novel tank
Latency to enter the top (s)	The amount of time to first cross from the bottom part to the top of the novel tank
Number of entries to the top	The number of crosses from the bottom part to the top of the novel tank
Time spent ratio of top: bottom	The ratio of the time spent on top over bottom
Distance travelled ratio of top: bottom	The ratio of the total distance moved in the top part vs. the bottom
Entries ratio of top: bottom	The number of crosses from the bottom part to the top of the novel tank
Freezing bouts (frequency)	The total number of instances of immobility (> 1 s) during the 5 min test in the novel tank
Freezing duration (s)	The duration of all freezing bouts in the novel tank

**Table 2:** The definitions of behavioural parameters in the maze

Behavioural parameters	Definition
Time (%)	The ratio of the time zebrafish spent in each arm (colour) to the total time spent in the maze
Distance (%)	The ratio of the distance zebrafish travelled in each arm (colour) to the total distance travelled in the maze

### Chemical and experimental design

Reserpine (purity ≥ 98.0%) was purchased from Shanghai Macklin Biomedical Co., Ltd. The reserpine concentration of 40 μg/mL in this study was chosen based on previous research concerning the effective doses of reserpine for the depressive behaviour of zebrafish [5, 45]. Sertraline hydrochloride (purity > 98.0%) was purchased from TCI Co., Ltd. (Shanghai, China). Preliminary experiments proved that the effective concentration of sertraline hydrochloride was 0.1 μg/mL. The experimental doses of reserpine (40 μg/mL) or sertraline hydrochloride (0.1 μg/mL) were obtained by weighing and adding dry powder to system water. JWXY capsule (Z10960066) was purchased from Sichuan Baoxing Pharmaceutical Co., LTD (Sichuang, China). The composition of JWXY capsule is as follows: *Bupleuri Radix, Angelicae Sinensis Radix, Paeoniae Radix Alba, Atractylodis Macrocephalae Rhizoma* (stir-baking with bran)*, Poria, Glycyrrhizae Radix Et Rhizoma, Menthae Haplocalycis Herba, Moutan Cortex,* and *Gardeniae Fructus* (processed with ginger juice). Based on preliminary experiments, the effective concentration of JWXY capsule was 100 μg/mL. We opened the capsule and grinded the dry powdered contents. Then, the powdered medicine was weighed and dissolved in system water to obtain a solution with a concentration of 100 μg/mL. Experimental solutions were sonicated for 30 min to dissolve the medication.

A total of 48 experimentally naïve, adult zebrafish (9 months old, male:female = 1:1) were used in our study. All zebrafish were housed in groups of 2 zebrafish per 4 L tank (filled with system water maintained at 28 °C) on a 14:10 h light cycle. The 48 zebrafish were first tested by 3D neurophenotyping in the novel tank and colour preference behaviour in the maze (defined as control). Then, all 48 zebrafish were exposed 40 μg/mL reserpine for 20 min and tested by behavioural apparatuses (defined as acute). Next, acute zebrafish were separated into three groups according to the experimental design. The three groups were exposed to system water (indicated as the model), 0.1 μg/mL sertraline hydrochloride (indicated as sertraline) and 100 μg/mL JWXY capsule (indicated as JWXY) for 7 days and then subjected to behavioural testing (Fig. 1a). Solutions were refreshed every day after feeding with fresh brine shrimp.

### Behavioural testing

All zebrafish used in our study were acclimated to the laboratory environment. Before the behavioural test at every endpoint, zebrafish were given 1 h to acclimate to the tank environment. Behavioural testing was performed between 9:00 am and 16:00 pm, i.e., the middle of the light phase of the light cycle, with tanks filled with system water at a temperature ranging from 26 to 28 °C. Zebrafish behaviours were recorded for 5 min by CCD cameras and evaluated by analysing the behavioural endpoints in Tables 1 and 2.

### Enzyme-linked immunosorbent assay (ELISA)

Cortisol was extracted from zebrafish whole-body homogenates. Adult zebrafish in different treatment groups were weighed and stored at − 80 °C. The whole zebrafish was dissected into small pieces on ice and homogenised in 500 μL ELISA Buffer, followed by sonication on ice for 30 s. Diethyl ether was added to samples, which were shaken for 10 min and centrifuged at 2000 rpm for 15 min at 4 °C. After storing the samples at − 80 °C for 15 min, the supernatant was transferred into new tubes. After the diethyl ether evaporated, the extracts were dissolved in 500 μL ELISA Buffer and analysed by using the Cortisol ELISA Kit (Cayman, 500360).

The NA, 5-HT and DA concentrations of adult zebrafish brains were analysed by a NA ELISA Kit (CUSABIO, Wuhan, China), 5-HT ELISA Kit (CUSABIO, Wuhan, China), and DA ELISA Kit (CUSABIO, Wuhan, China), respectively. Zebrafish brain tissue was rinsed with 1 × PBS, homogenised in 1 mL 1 × PBS and stored overnight at − 20 °C. After two freeze–thaw cycles, homogenates were centrifuged at 5000*g* for 5 min at 4 °C. The supernatant was transferred into new tubes and assayed immediately according to the manufacturer’s instruction.

### Western blot

Total protein was extracted from adult zebrafish brain tissue with radioimmunoprecipitation assay (RIPA) (CWBIO, Beijing, China) buffer containing phenylmethylsulfonyl fluoride (PMSF) (Sigma-Aldrich). Protein concentrations were quantified using a BCA Protein Assay Kit (CWBIO). Proteins were separated in 10% sodium dodecyl sulphate-polyacrylamide gel electrophoresis (SDS-PAGE) and transferred to a polyvinylidene fluoride (PVDF) membrane that was blocked with Tris-buffered saline (TBS) containing 5% skim milk for 1 h at room temperature. Membranes were incubated with mouse anti-TH (1:1000; Millipore) and mouse anti- glyceraldehyde 3-phosphate dehydrogenase (GAPDH) (1:5000; Proteintech) overnight primary antibodies at 4 °C. After being washed with TBS containing 0.05% Tween-20 (TBST), the membrane was incubated with anti-mouse HRP-conjugated secondary antibody (1:3000; CWBIO). The membrane was then washed with TBS containing 0.05% Tween-20, and Super Signal West Pico chemiluminescent substrate (Thermo Scientific) was used for detection.

### UPLC and Q-TOF-MS analyses

We opened the JWXY capsule and ground the dry powdered contents. The powdered medicine was dissolved in system water to obtain a solution. Next, we used the mixed solution for ultra-performance liquid chromatography (UPLC) and quadrupole time-of-flight mass spectrometry (Q-TOF-MS) analyses.

A Waters Acquity UPLC System (Waters, MA, USA) equipped with a photodiode array detector was used. The system was controlled by Masslynx V4.1 software (Waters Co.). An Acquity BEHC18 column (2.1 × 100 mm, 1.7 μm; Waters Co.) was used for separations. Using Rongchang capsule as an example, a gradient elution of 0.1% formic acid in water (A) and 0.1% formic acid in acetonitrile (B) was performed as follows: 2% B was obtained from 0 to 1 min, 2–10% B from 1 to 3 min, 10–15% B from 3 to 7 min, 15–30% B from 7 to 15 min, 30–50% B from 15 to 20 min, 50–80% B from 20 to 23 min, and 80–100% B from 23 to 24 min; In adition, 100% B was maintained from 24 to 25 min; 100–2% B was obtained from 25 to 27 min; and 2% B was maintained from 27 to 30 min. Other samples were slightly adjusted based on their ingredients and chemical polarity. The flow rate was 0.40 mL/min, and the column temperature was maintained at 35 °C. Accurate mass measurements and MS/MS were performed on a Waters Q-TOF Premier with an electrospray ionisation (ESI) system (Xevo G2-Q Tof, Waters MS Technologies, Manchester, UK). The electrospray ionisation mass spectrometry (ESI–MS) spectra were acquired in both the negative and positive ion voltage modes. The capillary voltages were set to 2.0 kV for the negative mode and 3.0 kV for the positive mode. The sample cone voltage was set to 40 V. High-purity nitrogen was used as the nebulisation and auxiliary gas. The nebulisation gas was set at a flow rate of 800 L/h at 450 °C, the cone gas was set at a flow rate of 50 L/h, and the source temperature was 120 °C. The Q-TOF Premieracquisition rate was 0.1 s, with a 0.2-s scan delay. The instrument was operated with the first resolving quadrupole in a wide pass mode (50–2000 Da) and with the collision cell operating at two alternative energies (i.e., 20 and 50 eV). Leucine enkephalin (200 pg/mL) was used as the lock mass ([M−H]^−^ 554.2615, [M+H]^+^556.2771).

### Data analyses

Data represents the mean ± SEM (standard error of the mean). One-way ANOVA was performed to assess differences between groups, followed by post hoc Tukey HSD tests for data with a normal distribution. A nonparametric Kruskal–Wallis test followed by Dunn’s multiple comparisons tests (*p < 0.05) was used for data that violated the assumption of normality. We used GraphPad Prism 7.0 to obtain statistical charts and graphs.

## Results

### Establishment of the experimental procedure and analyses of JWXY capsule constituents

All herbs in JWXY capsule are presented in Additional file 1: Table S1. We utilized a solution of capsule contents to conduct a novel tank assay and colour preference behavioural experiment. UPLC and Q-TOF-MS analyses were conducted for JWXY capsule analysis. Protonated [M+H]^+^ or deprotonated [M−H]^−^ ions were obtained with as much characteristic fragment information as possible to deduce the molecular and elemental compositions of every constituent. The inferred chemical structure was compared with published data and reported natural product information. The ESI–MS spectra were acquired in both the positive and negative ion voltage modes for each capsule. Here, we show the results of JWXY capsule in the positive and negative ion voltage mode (Fig. 1d). A total of 57 compounds were identified in JWXY capsule. Detailed identification results are presented in Additional file 1: Table S2, Figures S1–S8.

### Both sertraline and JWXY capsule rescued the decreased locomotive behaviour of zebrafish caused by reserpine

**Fig. 2:**
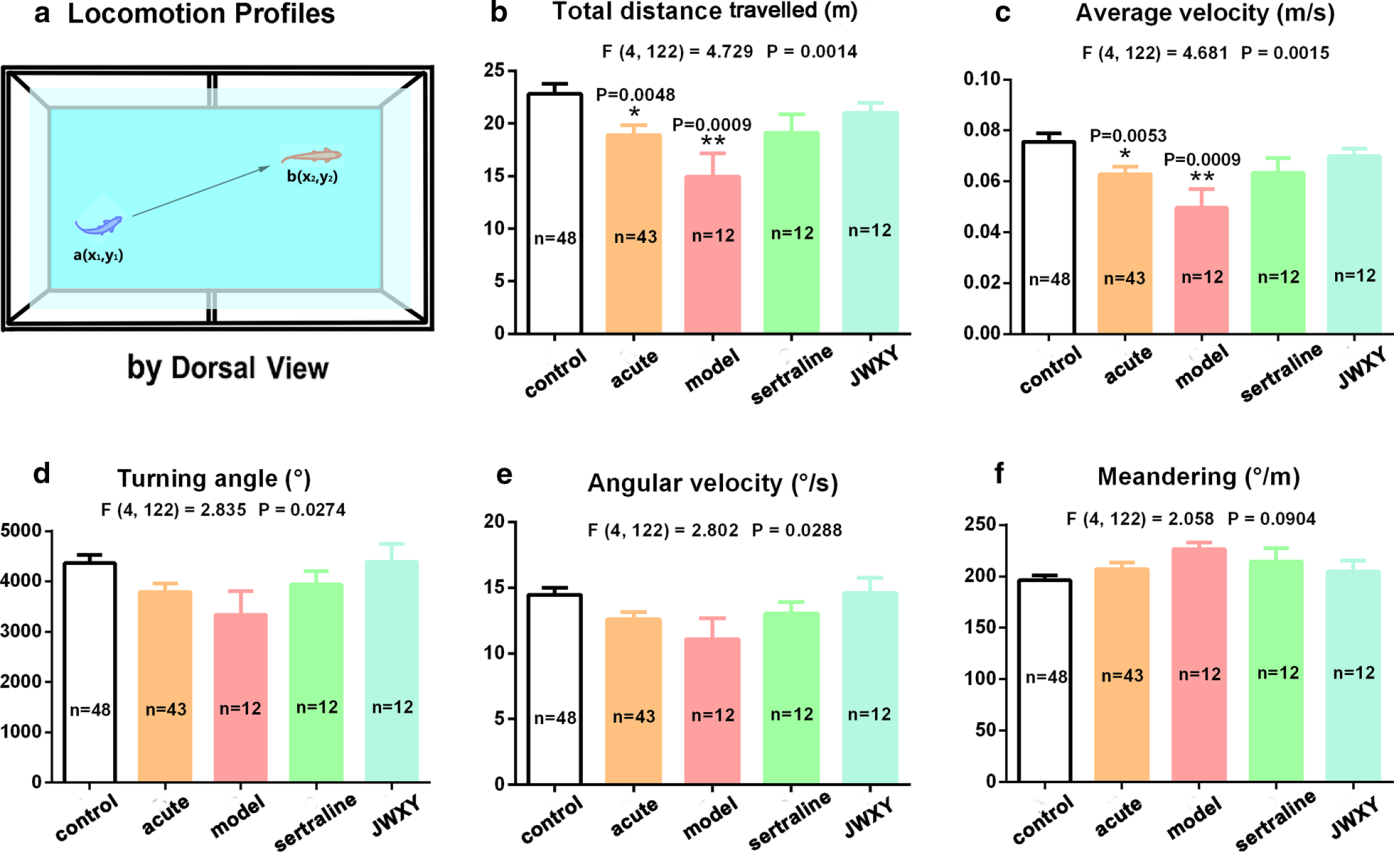
Locomotion profiles of zebrafish exposed to sertraline and JWXY capsule after reserpine treatment in the novel tank test. **a** Diagram of locomotion behaviour recorded by a camera from a dorsal view. Histogram revealing the locomotion behaviour of adult zebrafish by the **b** total distance travelled, **c** average velocity, **d** turn angle, **e** angular velocity and **f** meandering. Control: untreated AB strain zebrafish. Acute: acute treatment with reserpine for 20 min. Model: after acute treatment with reserpine, zebrafish were exposed to system water for 7 days to generate the depression model. Sertraline: after acute treatment with reserpine, zebrafish were exposed to sertraline for 7 days. JWXY: after acute treatment with reserpine, zebrafish were exposed to JWXY capsule for 7 days. The data are expressed as the mean ± S.E.M. and were analysed by one-way ANOVA followed by the Tukey post hoc test. Significance was defined as *p < 0.05, **p < 0.01

The locomotive behaviour of zebrafish was measured by the total distance travelled, average velocity, turn angle and angular velocity. Compared with the control, acute treatment (20 min) with reserpine resulted in suppression of the total distance travelled and average velocity. Then, after treatment with system water for 7 days, the total distance travelled and average velocity were more significantly reduced (model). The turn angle and angular velocity showed the same trend. However, total distance travelled was rescued after treatment with sertraline and JWXY capsule for 7 days (Fig. 2b). Moreover, the average velocity revealed that sertraline and JWXY capsule significantly rescued the reduced activity caused by reserpine (Fig. 2c). Likewise, the turn angle and angular velocity demonstrated that sertraline and JWXY capsule rescued the effects of reserpine on zebrafish behaviour (Fig. 2d, e). Reserpine slightly increased erratic movements measured by meandering in the novel tank test, and sertraline and JWXY capsule reduced this tendency (Fig. 2f).

### JWXY capsule rescued inhibition of exploratory behaviour and reversed the depressive phenotype of zebrafish

**Fig. 3:**
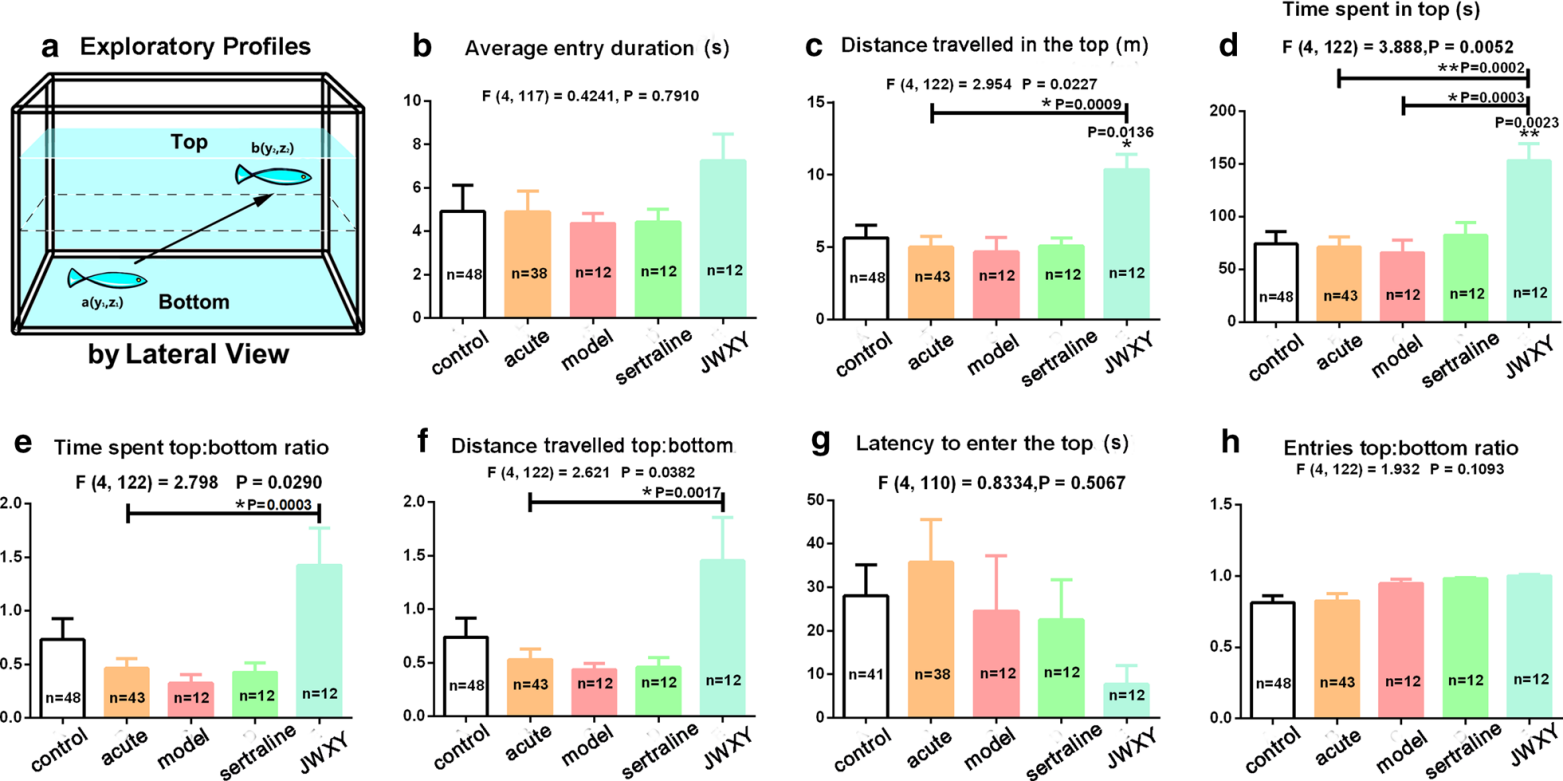
Exploratory profiles of zebrafish exposed to sertraline and JWXY capsule after reserpine treatment in the novel tank test. **a** Diagram of the exploratory behaviours recorded by a camera from a lateral view. The histograms demonstrated the exploratory behaviours of adult zebrafish by the **b** average entry duration to the top, **c** distance travelled in the top, **d** time spent in the top, **e** time spent top: bottom ratio, **f** distance travelled top: bottom, **g** latency to enter the top and **h** entries top: bottom ratio. Control: untreated AB strain zebrafish. Acute: acute treatment with reserpine for 20 min. Model: after acute treatment with reserpine, zebrafish were exposed to system water for 7 days to generate the depression model. Sertraline: after acute treatment with reserpine, zebrafish were exposed to sertraline for 7 days. JWXY: after acute treatment with reserpine, zebrafish were exposed to JWXY capsule for 7 days. The data are expressed as the mean ± S.E.M. and were analysed by one-way ANOVA followed by the Tukey post hoc test. Significance was defined as *p < 0.05, **p < 0.01

**Fig. 4:**
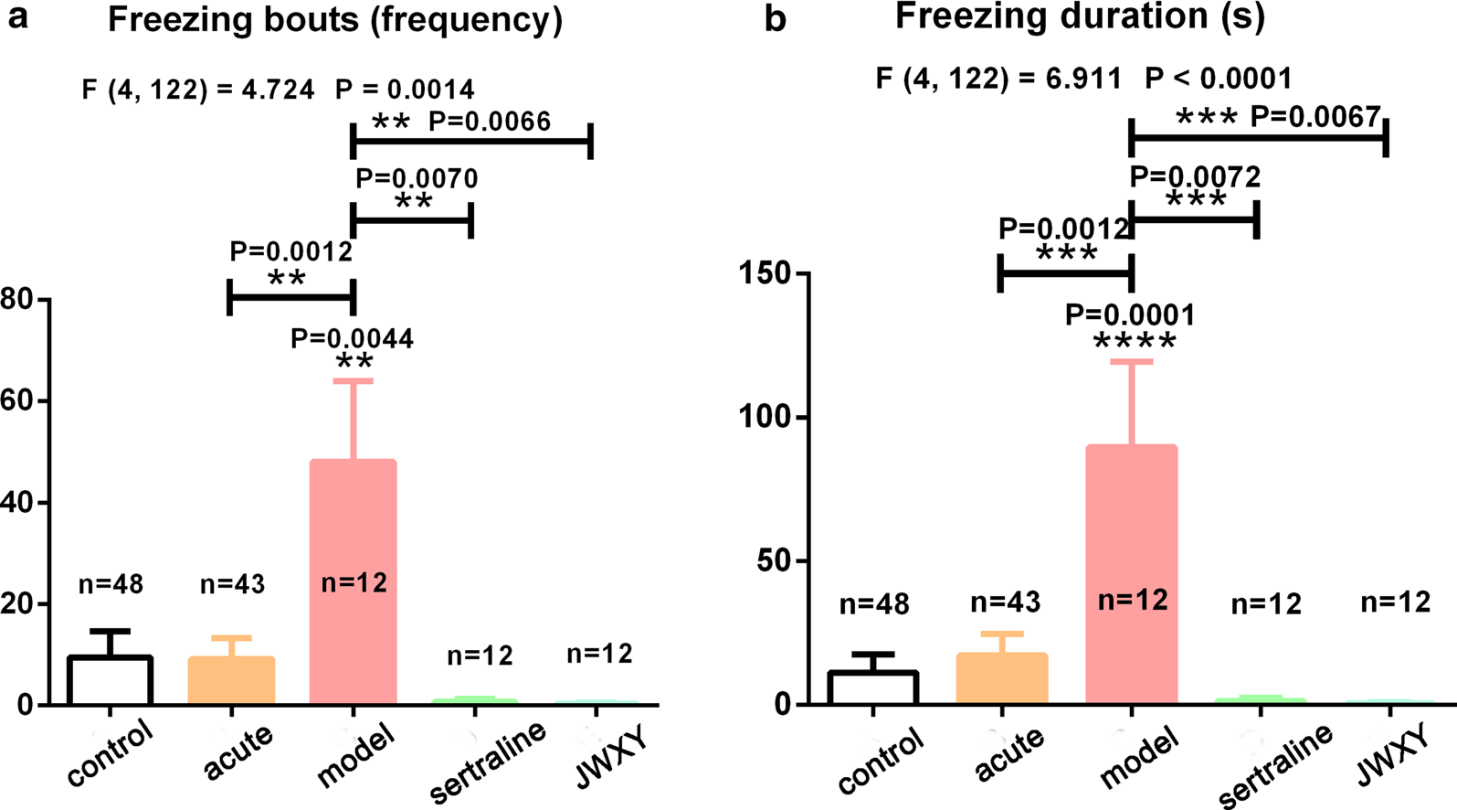
Histogram of the **a** freezing bouts and **b** freezing duration of zebrafish. Control: untreated AB strain zebrafish. Acute: acute treatment with reserpine for 20 min. Model: after acute treatment with reserpine, zebrafish were exposed to system water for 7 days to generate the depression model. Sertraline: after acute treatment with reserpine, zebrafish were exposed to sertraline for 7 days. JWXY: after acute treatment with reserpine, zebrafish were exposed to JWXY capsule for 7 days. The data are expressed as the mean ± S.E.M. and were analysed by one-way ANOVA followed by the Tukey post hoc test. Significance was defined as *p < 0.05, **p < 0.01, ***p < 0.001 and ****p < 0.0001

Exploratory behaviour, measured by the average entry duration (Fig. 3b), distance travelled in the top (Fig. 3c), time spent in the top (Fig. 3d), time spent ratio of top: bottom (Fig. 3e), distance travelled of top: bottom (Fig. 3f), latency to enter the top (Fig. 3g) and entries ratio of top: bottom (Fig. 3h), was not significantly altered in the treatment groups compared with that in the control group, with the exception of JWXY capsule treatment group. However, exploratory behavioural parameters were decreased in the model group. As shown in Fig. 3, zebrafish treated with JWXY capsule exhibited improvements in exploratory behaviour; the average entry duration, distance travelled in the top, time spent in the top, time spent ratio of top: bottom and distance travelled of top: bottom were significantly higher in the JWXY capsule group than in the acute group. Reserpine induced an obvious depressive phenotype as shown in Fig. 4. After acute treatment with reserpine, zebrafish did not show changes in their freezing bouts and freezing duration. However, the freezing bouts and freezing duration were enhanced after 7 days. After treatment with of sertraline and JWXY capsule for 7 days, the depressive phenotype was no longer observed. Moreover, the freezing bouts and freezing duration were significantly decreased.

### Impact of sertraline and JWXY capsule on the colour preference behaviour of zebrafish after reserpine treatment: JWXY capsule reversed colour preference patterns

**Fig. 5:**
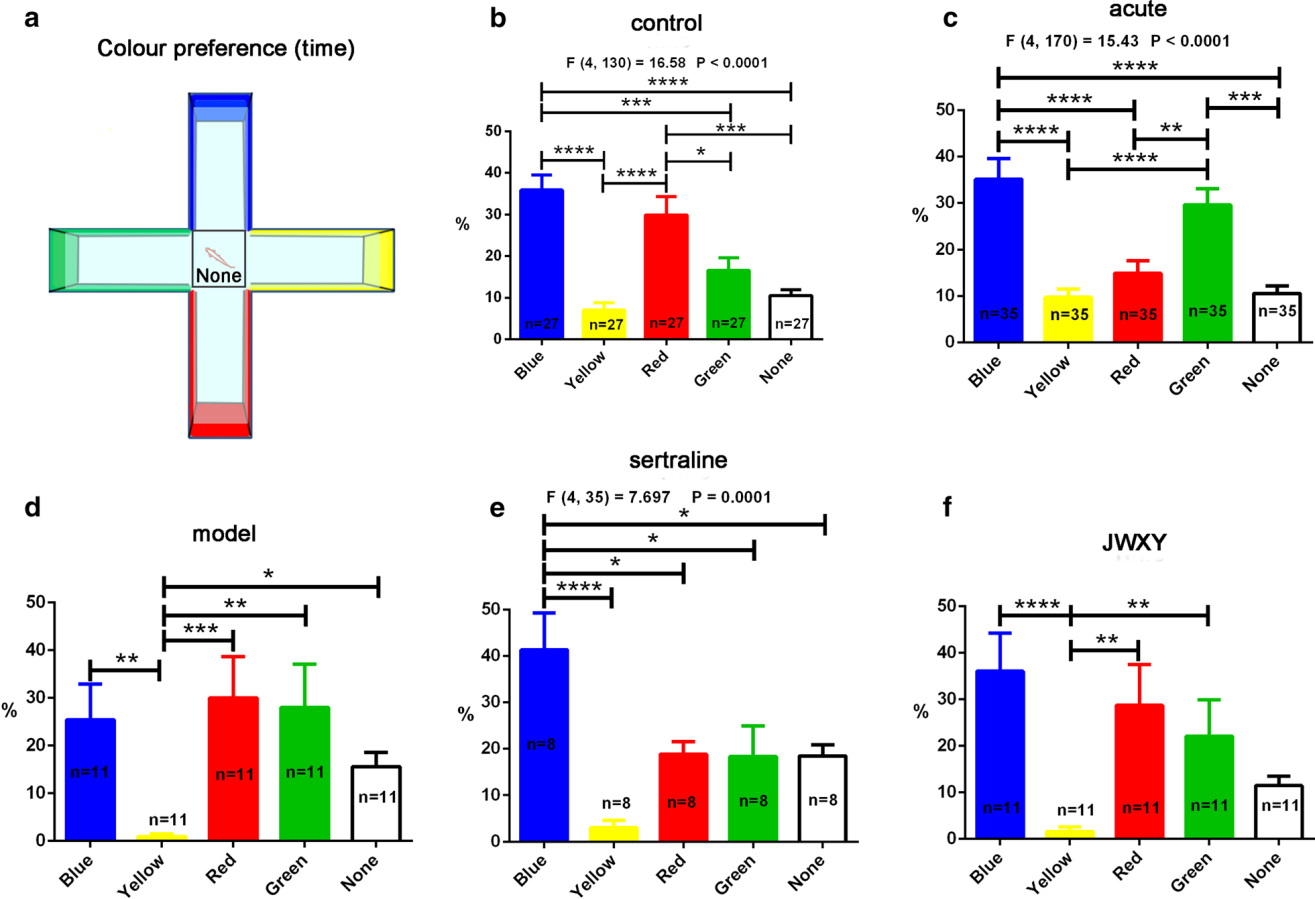
Colour preference profiles of zebrafish exposed to sertraline and JWXY capsule after reserpine treatment in the remoulded offset cross maze test. **a** Diagram of the remoulded offset cross maze and distribution of colours. The centre of the cross maze was denoted as None, and zebrafish started in that location. **b** The duration (time) of control zebrafish in every colour arm. **c** The duration (time) of zebrafish treated with reserpine for approximately 20 min (acute) in every colour arm. **d** The duration (time) of zebrafish exposed to system water after reserpine treatment (model) in every colour arm. **e** The duration (time) of zebrafish exposed to sertraline after reserpine treatment in every colour arm. **f** The duration (time) of zebrafish exposed to JWXY capsule after reserpine treatment at every colour arm. Control: untreated AB strain zebrafish. Acute: acute treatment with reserpine for 20 min. Model: after acute treatment with reserpine, zebrafish were exposed to system water for 7 days to generate the depression model. Sertraline: after acute treatment with reserpine, zebrafish were exposed to sertraline for 7 days. JWXY: after acute treatment with reserpine, zebrafish were exposed to JWXY capsule for 7 days. The data are expressed as the mean ± S.E.M. One-way ANOVA with post hoc Tukey HSD tests was used to analyse data with a normal distribution, and a nonparametric Kruskal–Wallis test followed by Dunn’s multiple comparisons tests was used for data that violated the assumption of normality. Significance was defined as *p < 0.05, **p < 0.01, ***p < 0.001 and ****p < 0.0001

Colour preference behaviour was demonstrated by the time spent (Fig. 5) and distance travelled (Additional file 1: Figure S9) in every colour arm using a remoulded offset cross maze (Fig. 5a). The control group (Fig. 5b) spent the most time in the blue area, followed by the red, green, and yellow areas (listed from most to least time spent). The most and least preferred colours were unchanged, but the time spent in green area was higher than that in the red area in the acute group (Fig. 5c). In the model group (Fig. 5d), the amounts of time spent in blue, red and green were not significantly different. Zebrafish continued to spend the least amount of time in the yellow area. After sertraline treatment (Fig. 5e), zebrafish recovered their preference for blue. However, the time spent in the red and green areas was not different. Notably, the JWXY group and control group had the same colour preference (Fig. 5f). The distance (Additional file 1: Figure S9) travelled in every colour arm was the same over time. Based on the abovementioned results, we concluded that zebrafish preferred blue the most and yellow the least. Following treatment with sertraline and JWXY capsule, the colour preference order was restored, and JWXY capsule was more effective than sertraline in restoring colour preference behaviour.

**Fig. 6:**
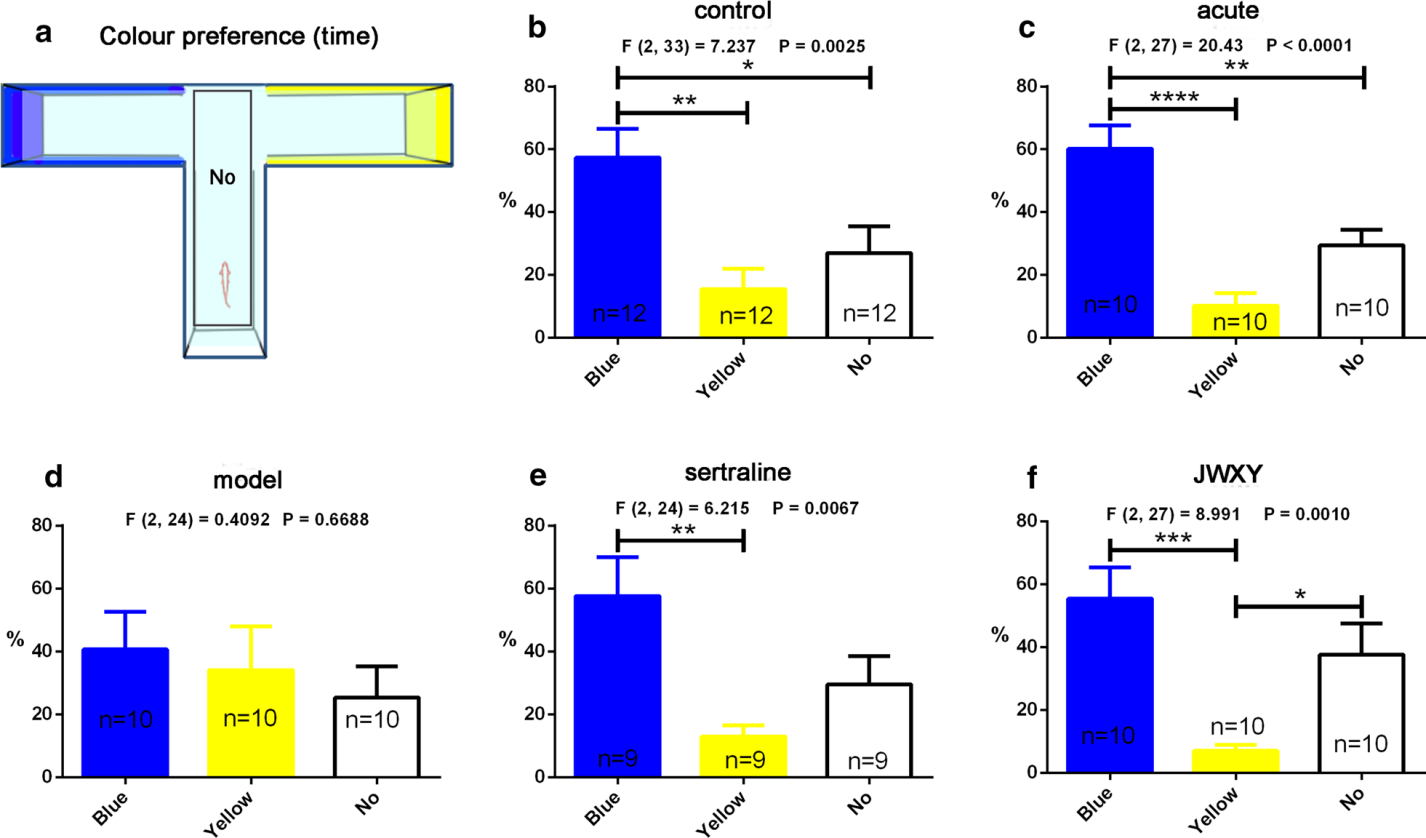
Colour preference profiles of zebrafish exposed to sertraline and JWXY capsule after reserpine treatment in the T-maze test. **a** Diagram of the T-maze and distribution of colours. The centre of the T-maze was denoted as No, and zebrafish started in that location. **b** The duration (time) of WT zebrafish in every colour arm. **c** The duration (time) of zebrafish treated with reserpine for approximately 20 min (acute) in every colour arm. **d** The duration (time) of zebrafish exposed to system water (model) after reserpine treatment in every colour arm. **e** The duration (time) of zebrafish exposed to sertraline after reserpine treatment in every colour arm. **f** The duration (time) of zebrafish exposed to JWXY capsule after reserpine treatment in every colour arm. Control: untreated AB strain zebrafish. Acute: acute treatment with reserpine for 20 min. Model: after acute treatment of reserpine, zebrafish were exposed to system water for 7 days to generate the depression model. Sertraline: after acute treatment with reserpine, zebrafish were exposed to sertraline for 7 days. JWXY: after acute treatment with reserpine, zebrafish were exposed to JWXY capsule for 7 days. The data are expressed as the mean ± S.E.M. and were analysed by one-way ANOVA followed by the Tukey post hoc test. Significance was defined as *p < 0.05, **p < 0.01, ***p < 0.001 and ****p < 0.0001

Based on colour preference behaviour in the remoulded offset cross maze, we chose two colours (blue and yellow) to conduct a concise test using a T-maze. Colour preference behaviour was demonstrated by time (Fig. 6) and distance (Additional file 1: Figure S10) travelled in every colour arm using a T-maze (Fig. 6a). In the control (Fig. 6b) and acute (Fig. 6c) groups, zebrafish spent significantly more time in the blue area than in the yellow area. However, the time zebrafish spent in every colour arm was not different in the model group (Fig. 6d). After sertraline (Fig. 6e) and JWXY capsule (Fig. 6f) treatment, the same colour preference tendency as that of the control was observed. The distance (Additional file 1: Figure S10) travelled in every colour arm was consistent with the time spent.

### Cortisol and monoamine levels influenced zebrafish neurobehaviour

**Fig. 7:**
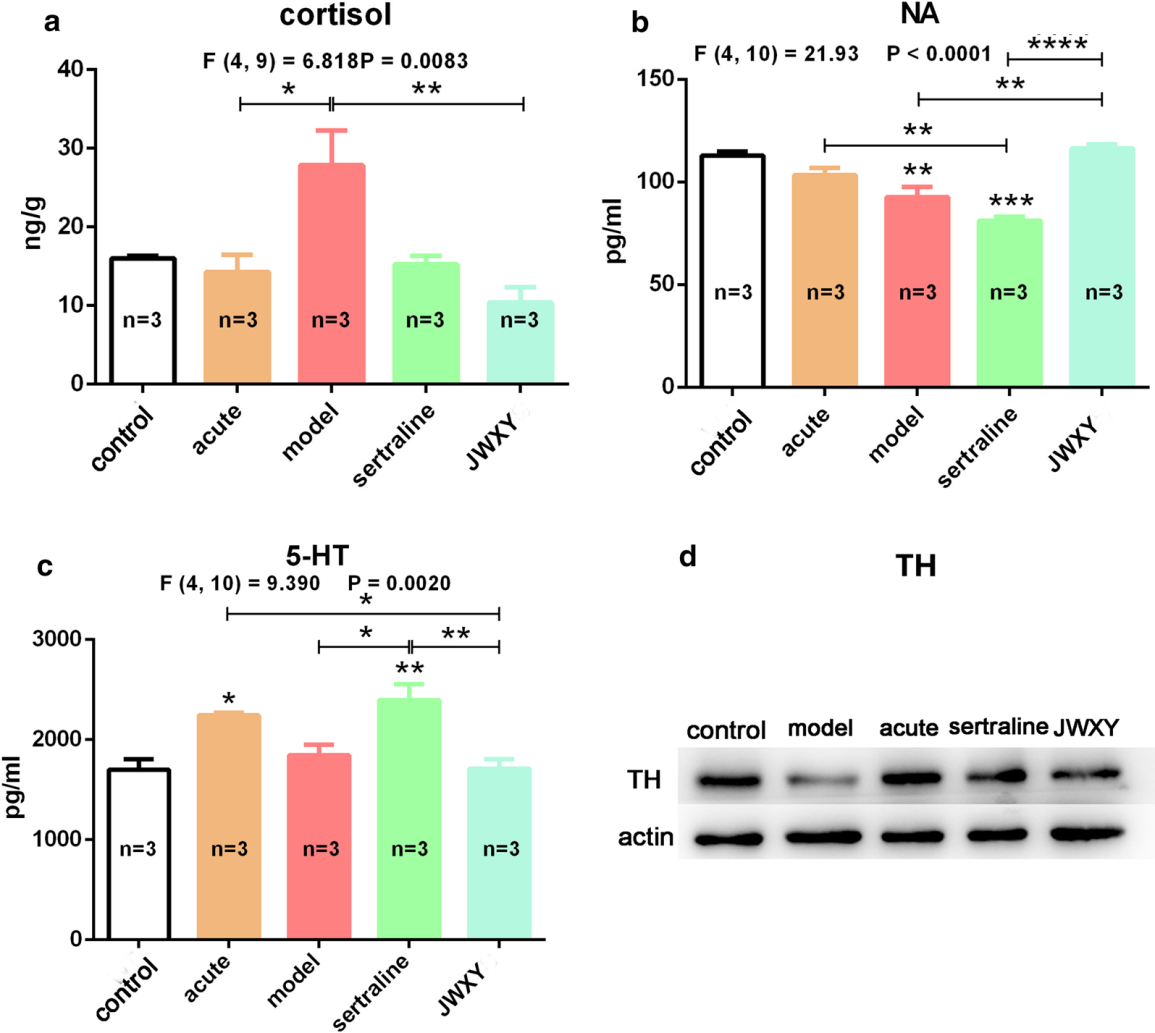
The effects of sertraline and JWXY capsule treatment on cortisol and monoamines in zebrafish. The levels of cortisol (**a**), noradrenaline (**b**), 5-HT (**c**) and tyrosine hydroxylase (**d**) in zebrafish after different treatments. Control: untreated AB strain zebrafish. Acute: acute treatment with reserpine for 20 min. Model: after acute treatment with reserpine, zebrafish were exposed to system water for 7 days to generate the depression model. Sertraline: after acute treatment with reserpine, zebrafish were exposed to sertraline for 7 days. JWXY: after acute treatment with reserpine, zebrafish were exposed to JWXY capsule for 7 days. The data are expressed as the mean ± S.E.M. and were analysed by one-way ANOVA followed by the Tukey post hoc test. Significance was defined as *p < 0.05, **p < 0.01, ***p < 0.001 and ****p < 0.0001

Whole-body cortisol, monoamines, including NA, 5-HT, DA, and TH were detected in zebrafish brain tissues after different treatments. The cortisol level in the model group was significantly higher than that in the acute and control groups. After sertraline and JWXY capsule treatments, the cortisol level was markedly decreased, especially in the JWXY group (Fig. 7a). NA in the model group was significantly lower than that in the control and sertraline group. However, compared with the model and sertraline groups, the JWXY group showed a significant increase in the NA concentration (Fig. 7b). 5-HT was significantly increased in the acute group but increased only slightly in the model group. Sertraline elevated the 5-HT level, but JWXY capsule did not (Fig. 7c). Sertraline decreased TH expression 7 days after acute exposure, and sertraline and JWXY capsule treatments improved TH to a degree (Fig. 7d). However, the DA level did not change after sertraline and JWXY treatments (Additional file 1: Figure S11).

## Discussion

JWXY capsule contains nine herbal medicines, and 57 compounds were identified in its extraction by UPLC and Q-TOF-MS. As previously described, TCM posits that depression involves in multiple organs. TCM focuses on the overall effect of medicines contained in a prescription, and it also plays a role in health care and disease prevention through the treatment of multiple targets. All herbal medicines in the prescription work synergistically and can yield stable and comprehensive curative effects, greatly reducing the side effects of drug treatment. Determining the main components of TCM prescriptions and their mechanisms of action is difficult. However, characterising the multiple constituents, targets and pathways of TCM prescriptions is of greater importance, and requires further research.

We employed a 3D video-tracking system to detect changes in the swimming behaviour of lesioned zebrafish in novel tank. Zebrafish demonstrated long-term depressive symptoms, including elevated baseline whole-body cortisol, social withdrawal and locomotor retardation after reserpine exposure [46]. Reserpine does not induce overt acute behavioural effects but markedly reduces activity after 7 days [5], consistent with our study. After 20 min of reserpine exposure, zebrafish showed a slight decrease in locomotive activity but did not show obvious changes in exploratory behaviour and freezing behaviour. Zebrafish displayed significantly decreased locomotive activity and a worsened depressive phenotype after 7 days, along with hypoactive exploratory behaviour, which proved that the establishment of zebrafish depression model first introduced by Kyzar et al. was successful in our experiment. However, compared with zebrafish in the model group, zebrafish in the sertraline and JWXY capsule groups treated for 7 days expressed different behaviours. Sertraline treatment increased locomotive activity and rescued the depressive phenotype induced by reserpine. Moreover, JWXY capsule increased locomotive activity, more effectively reversed the depressive phenotype, and improved exploratory behaviour.

The colour preference test could serve as a useful protocol for memory evaluation, cognitive dysfunction, assessment of neurodegenerative disorders, preclinical appraisal of drug efficacy and behavioural evaluation of toxicity [47]. Here, we evaluated cognitive impairment by the colour preference test. Studies have demonstrated the natural colour preference of zebrafish. Zebrafish prefer colours of short wavelengths. Zebrafish exhibit a strong preference for blue relative to all other colours (red, yellow and green), with yellow being less preferred than red and green [26, 48]. In our study, blue was the favourite colour of control zebrafish, and yellow was the least favourite. Control zebrafish exhibited a significantly stronger preference for blue than for red and green. However, compared with control zebrafish, zebrafish exhibiting depressive behaviour lost certain colour preferences. Yellow was the least preferred colour of model zebrafish, but the preference for green and red increased simultaneously and was not significantly different compared with that for blue, indicating that the normal colour preference pattern was disturbed. However, Zebrafish treated by JWXY capsule regained this colour preference pattern. Sertraline also restored the colour preference pattern to a degree, but its efficacy was not as obvious and clear as that of JWXY capsule. To minimize the effects of place preference on the results and further verify this preference in zebrafish, we chose to test blue and yellow in T-maze. All groups except the model group exhibited a preference for blue. However, different from the other groups, the model group also showed an increased preference for yellow and the same preference for all three arms, illustrating the colour preference disorder in depressed zebrafish. In contrast, sertraline and JWXY capsule restored the colour preference pattern. These results showed that the cognitive dysfunction accompanying with depression in zebrafish could be reversed by sertraline and JWXY capsule.

Depression is usually comorbid with anxiety, which leads to behavioural alterations. The effects of chronic depression and anxiety on the hypothalamic–pituitary–interrenal (HPI) axis have been studied previously in zebrafish. Benzodiazepines (anxiolytics) and antidepressants completely prevent increased cortisol levels in zebrafish [49]. The decreases in total distance travelled and velocity in zebrafish are related to the decreased levels of DA and NA [50]. SSRIs were developed and entered clinical trials as a new class of antidepressant in the 1980s. Six SSRIs, including fluoxetine, paroxetine, sertraline, fluvoxamine, citalopram and escitalopram are commonly used for clinical treatment. SSRIs selectively inhibit the reuptake of 5-HT by the presynaptic membrane. SSRIs have little impact on NA and hardly affect the reuptake of DA [51]. In our study, the reserpine-induced zebrafish model of depression showed increased whole-body cortisol and 5-HT, decreased NA and reduced TH. Compared with the model, sertraline prevented the increase in cortisol and NA and increased 5-HT and TH. However, JWXY capsule prevented the increase in cortisol and 5-HT, consistent with the rescued depressive phenotype. In addition, compared with the model, JWXY capsule improved the levels of NA and TH, consistent with the increased locomotive activity. Interestingly, DA levels in zebrafish brains were unaffected by any treatments. Those changes in monoamine neurotransmitters were related to the colour preference disorder caused by reserpine and were consistent with the restored cognitive ability.

## Conclusion

The novel tank test recorded by a 3D method in this experiment revealed the similar anti-depression effects of two treatments for chronic reserpine exposure. This validation was based on the successful establishment of a depressive zebrafish model, which was first introduced by Kyzar et al. The depressive effects of reserpine decrease locomotion, increase erratic movements, reduce exploratory behaviour to the top and enhance depressive phenotype. Furthermore, colour preference testing in a remoulded offset cross maze and T-maze indicated that the natural colour preference pattern (zebrafish prefer blue to red, green and yellow and show a strong aversion to yellow) was disturbed due to depression induced by reserpine. However, sertraline treatment improved depression-like behaviours by increasing locomotion and decreasing erratic movements and the depressive phenotype. Sertraline also restored the colour preference in zebrafish. Notably, JWXY capsule was a more effective treatment than sertraline. JWXY capsule treatment reversed depression-like behaviours by increasing locomotion, decreasing erratic movements, increasing exploratory behaviour to the top and rescued the depressive phenotype. Zebrafish also exhibited their natural colour preference after JWXY capsule treatment.

Depression-like behaviours and cognitive disorder (measured by colour preference) resulted from changes in hormone and monoamine neurotransmitters in the brain. Increased whole-body cortisol and decreased NA and TH were observed in the zebrafish depression model. Sertraline prevented the increase in cortisol, inhibited the reuptake of 5-HT, and improved the expression of TH. Compared with the model, JWXY capsule also prevented the increase in cortisol, recovered NA and improved the expression of TH. Overall, these results show that changes in cortisol and monoamines accounted for the reversal of depressive behaviours and cognitive dysfunction. The high sensitivity of zebrafish to the effects of Western medicine and TCM can help improve our understanding of the psychopharmacological profiles of these drugs and related CNS drugs, as well contribute to further development of TCM as an antidepressant.

## Additional file

**Additional file 1: Table S1.** The composition of JWXY capsule. **Figure S1.** ESI-MS spectra in the positive and negative ion voltage mode of JWXY capsule (1–30 min). **Figure S2.** ESI-MS spectra in the positive and negative ion voltage mode of JWXY capsule (15-30min). **Table S2.** MS data in (±) ESI modes and the identification results in JWXY capsule. **Figures S3**–**8.** The chemical structure of each component identified in JWXY capsule. **Figure S9.** Colour preference profiles of zebrafish exposed to sertraline and JWXY capsule after reserpine treatment in the remoulded offset cross maze test. **Figure S10.** Colour preference profiles of zebrafish exposed to sertraline and JWXY capsule after reserpine treatment in the T-maze test. **Figure S11.** The changes of sertraline and JWXY capsule treatment on dopamine (DA) of zebrafish.
