# Supplementary material for: Reversal of reserpine-induced depression and cognitive disorder in zebrafish by sertraline and Traditional Chinese Medicine (TCM)
Source: Behav Brain Funct. 2018 Jun 14;14:13. doi: 10.1186/s12993-018-0145-8 (PMC6001006; doi:10.1186/s12993-018-0145-8)
Supplement: Supplementary file 1 — Additional file 1: Table S1. The composition of JWXY capsule. Figure S1. ESI-MS spectra in the positive and negative ion voltage mode of JWXY capsule (1–30 min). Figure S2. ESI-MS spectra in the positive and negative ion voltage mode of JWXY capsule (15-30min). Table S2. MS data in (±) ESI modes and the identification results in JWXY capsule. Figures S3–8. The chemical structure of each component identified in JWXY capsule. Figure S9. Colour preference profiles of zebrafish exposed to sertraline and JWXY capsule after reserpine treatment in the remoulded offset cross maze test. Figure S10. Colour preference profiles of zebrafish exposed to sertraline and JWXY capsule after reserpine treatment in the T-maze test. Figure S11. The changes of sertraline and JWXY capsule treatment on dopamine (DA) of zebrafish. [file 12993_2018_145_MOESM1_ESM.docx]

Additional file

**Reversal of reserpine-induced depression and cognitive disorder in zebrafish**

**by sertraline and traditional Chinese medicine (TCM)**

Shuhui Zhang ^a,1^, Xiaodong Liu ^a,1^, Mingzhu Sun ^c,1^, Qiuping Zhang ^b,1^, Teng Li ^c^, Xiang Li ^a^, Jia Xu ^b^, Xin Zhao ^c,*^, Dongyan Chen ^b,*^, Xizeng Feng ^a,*^

^a^State Key Laboratory of Medicinal Chemical Biology, The Key Laboratory of Bioactive Materials, Ministry of Education. College of Life Science, Nankai University, Tianjin 300071, China.

^b^Tianjin Key Laboratory of Tumor Microenvironment and Neurovascular Regulation, Department of Histology and Embryology, School of Medicine, Nankai University, Tianjin 300071, China.

^c^The Institute of Robotics and Automatic Information Systems, Nankai University, Tianjin 300071, China.

^1^These authors contributed equally to this work.

^*^Correspondence and requests for materials should be addressed to X.Z. (email: [zhaoxin@nankai.edu.cn](mailto:zhaoxin@nankai.edu.cn)), D.Y.C. (email: chendy@nankai.edu.cn), or X.Z.F. (email: xzfeng@nankai.edu.cn)

**Table of Contents**

1. **Additional file Tables and Figures**
   1. **Table S1.** The composition of JWXY capsule.
   2. **Figure S1.** ESI-MS spectra in the positive and negative ion voltage mode of JWXY capsule (1-30 min).
   3. **Figure S2.** ESI-MS spectra in the positive and negative ion voltage mode of JWXY capsule (15-30min).
   4. **Table S2.** MS data in (±) ESI modes and the identification results in JWXY capsule.
   5. **Figure S3-8.** The chemical structure of each component identified in JWXY capsule.
   6. **Figure S9.** Colour preference profiles of zebrafish exposed to sertraline and JWXY capsule after reserpine treatment in the remoulded offset cross maze test.
   7. **Figure S10.** Colour preference profiles of zebrafish exposed to sertraline and JWXY capsule after reserpine treatment in the T-maze test.
   8. **Figure S11.** The changes of sertraline and JWXY capsule treatment on dopamine (DA) of zebrafish.

**Table S1. The composition of JWXY capsule**

| **Name** | **Latin Name** |
| --- | --- |
| Chinese Thorowax Root | *Bupleuri Radix* |
| Chinese Angelica | *Angelicae Sinensis Radix* |
| White Paeony Root | *Paeoniae Radix Alba,* |
| Largehead Atractylodes Rhizome | *Atractylodis Macrocephalae Rhizoma* |
| Indian Buead | *Poria* |
| Lithargite Root | *Glycyrrhizae Radix Et Rhizoma* |
| Peppermint | *Menthae Haplocalycis Herba* |
| Tree Peony Bark | *Moutan Cortex* |
| Cape Jasmine Fruit | *Gardeniae Fructus* |


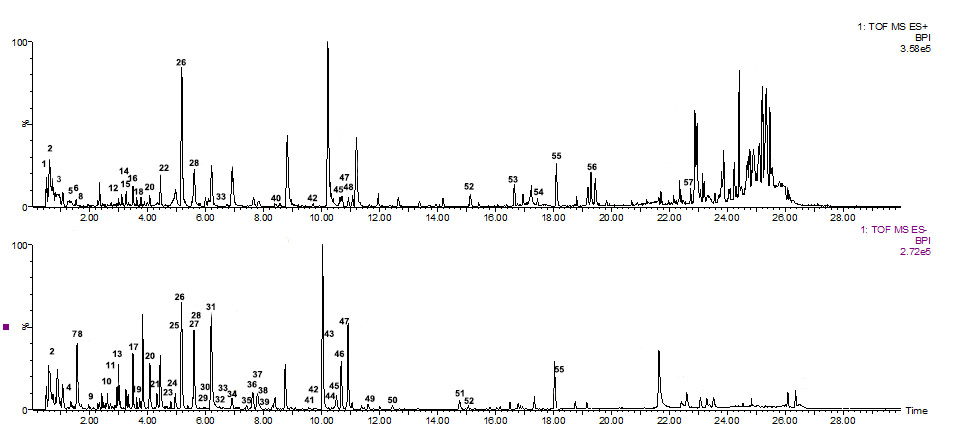


**Figure S1. ESI-MS spectra in the positive and negative ion voltage mode of JWXY capsule (1-30 min).** Some of the constituents are labelled in the spectra.


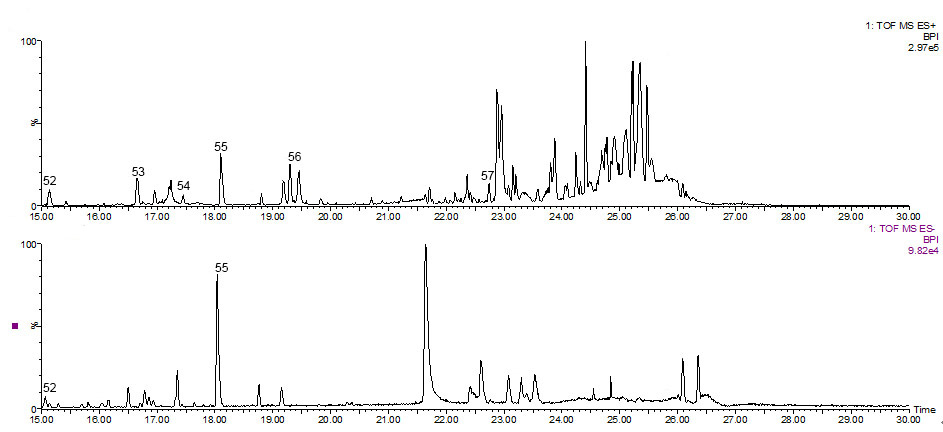


**Figure S2. ESI-MS spectra in the positive and negative ion voltage mode of JWXY capsule (15-30 min).** Some of the constituents are labelled in the spectra.

**Table S2.** **MS data in (±) ESI modes and the identification results in JWXY capsule**

| Peak No. | tR(min) | Identification | Pos (m/z) | Neg (m/z) | Molecular formula | Herb |
| --- | --- | --- | --- | --- | --- | --- |
| 1 | 0.59 | Valine | 118.0892[M+H]^+^ |  | C_5_H_11_NO_2_ | PT |
| 2 | 0.63 | Sucrose | 343.1259[M+H]^+^ | 341.1069[M+H]^-^ | C_12_H_22_O_11_ | CM |
| 3 | 0.94 | Tyrosine | 182.0856[M+H]^+^ |  | C_9_H_11_NO_3_ | PT |
| 4 | 1.25 | Succinic acid |  | 117.0193[M+H]^-^ | C_4_H_6_O_4_ | RAS |
| 5 | 1.36 | Paeonilactone B | 197.0817[M+H]^+^ |  | C_10_H_12_O_4_ | RPA |
| 6 | 1.47 | Adenine | 136.0637[M+H]^+^ |  | C_5_H_5_N_5_ | RAS |
| 7 | 1.56 | Gallic acid |  | 169.014[M+H]^-^ | C_7_H_6_O_5_ | RPA |
| 8 | 1.56 | 5-Hydroxymethyl-2-furfural | 149.0242[M+Na]^+^ | 125.0242[M+H]^-^ | C_6_H_6_O_3_ | RPA |
| 9 | 2.08 | Glucogallin |  | 331.0657[M+H]^-^ | C_13_H_16_O_10_ | RPA |
| 10 | 2.73 | Caffeic acid |  | 179.0333[M+H]^-^ | C_9_H_8_O_4_ | PT |
| 11 | 2.87 | Mudanoside A |  | 329.0861[M+H]^-^ | C_14_H_18_O_9_ | CM |
| 12 | 2.99 | Hemiarin | 177.0565[M+H]^+^ |  | C_10_H_8_O_3_ | RB |
| 13 | 3.09 | Lactinolide |  | 199.0975[M+H]^-^ | C_10_H_16_O_4_ | RPA |
| 14 | 3.25 | Limettin(Citropten) | 207.0667[M+H]^+^ |  | C_11_H_10_O_4_ | RB |
| 15 | 3.34 | Umbelliferone | 163.04[M+H]^+^ |  | C_9_H_6_O_3_ | RGH |
| 16 | 3.46 | Gardenoside | 405.1417[M+H]^+^ |  | C_17_H_24_O_11_ | GJE |
| 17 | 3.57 | Mudanpioside F/G |  | 343.138[M+H]^-^ | C_16_H_24_O_8_ | CM |
| 18 | 3.63 | Scoparone | 207.0662[M+H]^+^ |  | C_11_H_10_O_4_ | RB |
| 19 | 3.73 | Paeonol |  | 165.0562[M+H]^-^ | C_9_H_10_O_3_ | RPA |
| 20 | 4.07 | Chlorogenic acid | 355.1046[M+H]^+^ | 353.0882[M+H]^-^ | C_16_H_18_O_9_ | GJE |
| 21 | 4.32 | 5,6,4'-Trihydroxy-7,8-dimethoxyflavone  /5,8,4'-Trihydroxy-6,7-dimethoxyflavone |  | 375.0721[M+CH2O2^-^]^-^ | C_17_H_14_O_7_ | PT |
| 22 | 4.45 | Genipin | 227.0935[M+H]^+^ |  | C_11_H_14_O_5_ | GJE |
| 23 | 4.55 | 4-Methoxy-3-hydroxybenzoic acid |  | 167.0351[M+H]^-^ | C_8_H_8_O_4_ | CM |
| 24 | 4.74 | Isoliquiritin  /Neoisoliquiritin |  | 417.1194[M+H]^-^ | C_21_H_22_O_9_ | RGH |
| 25 | 5.09 | Oxypaeoniflorin |  | 495.1507[M+H]^-^ | C_23_H_28_O_12_ | RPA |
| 26 | 5.17 | Geniposide | 389.1454[M+H]^+^ | 433.1357[M+CH2O2^-^]^-^ | C_17_H_24_O_10_ | GJE |
| 27 | 5.56 | Paeonolide |  | 459.153[M+H]^-^ | C_20_H_28_O_12_ | CM |
| 28 | 5.61 | Albiflorin | 481.1698[M+H]^+^ | 479.1566[M+H]^-^ | C_23_H_28_O_11_ | RPA |
| 29 | 5.99 | 1-O-β-D-Glucopyranosylpaeonisuffrone |  | 375.1658[M+H]^-^ | C_17_H_28_O_9_ | RPA |
| 30 | 6.09 | (+)-Catechin |  | 335.0773[M+CH2O2^-^]^-^ | C_15_H_14_O_6_ | CM |
| 31 | 6.2 | Mudanpioside E |  | 525.1618[M+H]^-^ | C_24_H_30_O_13_ | CM |
| 32 | 6.42 | Suffruticoside B |  | 611.1637[M+H]^-^ | C_27_H_32_O_16_ | CM |
| 33 | 6.6 | Ferulic acid | 195.0658[M+H]^+^ | 193.0509[M+H]^-^ | C_10_H_10_O_4_ | RGH |
| 34 | 6.9 | poricoic acid A/F |  | 497.3277[M+H]^-^ | C_31_H_46_O_5_ | PR |
| 35 | 7.45 | Isoliquiritigenin /Liquiritigenin |  | 255.0634[M+H]^-^ | C_15_H_12_0_4_ | RGH |
| 36 | 7.65 | Kaempferol-3,7-di-O-β-D-glucoside |  | 609.1415[M+H]^-^ | C_27_H_30_O_16_ | RPA |
| 37 | 7.8 | Licurazide |  | 549.1569[M+H]^-^ | C_26_H_30_O_13_ | RGH |
| 38 | 7.88 | Anisic acid |  | 151.0389[M+H]^-^ | C_8_H_8_O_3_ | RAS |
| 39 | 8.06 | Suffruticoside A |  | 611.1550[M+H]^-^ | C_27_H_32_O_16_ | CM |
| 40 | 8.44 | Galloylpaeoniflorin | 655.1663[M+Na]^+^ |  | C_30_H_32_O_15_ | CM |
| 41 | 9.61 | 2-Ethyl-2-hexenoic aldehyde |  | 125.0964[M+H]^-^ | C_8_H_14_O | GJE |
| 42 | 9.79 | Paeoniflorin | 481.1698[M+H]^+^ | 479.1560[M+H]^-^ | C_23_H_28_O_11_ | CM |
| 43 | 10.1 | Diosmin |  | 607.1675[M+H]^-^ | C_28_H_32_O_15_ | PT |
| 44 | 10.4 | Mudanpioside D |  | 509.166[M+H]^-^ | C_24_H_30_O_12_ | CM |
| 45 | 10.52 | Mudanpioside I | 481.1698[M+H]^+^ | 479.1558[M+H]^-^ | C_23_H_28_O_11_ | CM |
| 46 | 10.75 | Benzoyloxy  paeoniflorin |  | 599.1760[M+H]^-^ | C_30_H_32_O_13_ | CM |
| 47 | 10.8 | Lactiflorin | 463.1582[M+H]^+^ | 461.1445[M+H]^-^ | C_23_H_26_O_10_ | RPA |
| 48 | 10.92 | d-Catechin | 291.0903[M+H]^+^ |  | C_15_H_14_O_6_ | RPA |
| 49 | 11.64 | Mudanpioside C |  | 599.1771[M+H]^-^ | C_30_H_32_O_13_ | CM |
| 50 | 12.45 | Oxybenzoylpaeoniflorin |  | 599.1760[M+H]^-^ | C_30_H_32_O_13_ | RPA |
| 51 | 14.77 | Mudanpioside J |  | 629.1894[M+H]^-^ | C_31_H_34_O_14_ | CM |
| 52 | 15.07 | Benzoylpaeoniflorin | 585.2006[M+H]^+^ | 583.1836[M+H]^-^ | C_30_H_32_O_12_ | RPA |
| 53 | 16.68 | Isoglabrolide | 469.3351[M+H]^+^ |  | C_30_H_44_O_4_ | RGH |
| 54 | 17.44 | Glabrolide | 469.3339[M+H]^+^ |  | C_30_H_44_O_4_ | RGH |
| 55 | 18.1 | Glycyrrhizic acid | 823.4152[M+H]^+^ | 821.3985[M+H]^-^ | C_42_H_62_O_16_ | RGH |
| 56 | 19.36 | Atractylenolide I | 231.1367[M+H]^+^ |  | C_15_H_18_O_2_ | RAM |
| 57 | 22.69 | Brefeldin A | 281.1746[M+H]^+^ |  | C_16_H_24_O_4_ | RAS |

PT: *Menthae Haplocalycis Herba*; CM: *Moutan Cortex*; RAS: *Angelicae Sinensis Radix*; RPA: *Paeoniae Radix Alba*; RB: *Bupleuri Radix*; RGH: *Glycyrrhizae Radix Et Rhizoma*; GJE: *Gardeniae Fructus*; PR: *Poria*; RAM: *Atractylodis Macrocephalae Rhizoma*.


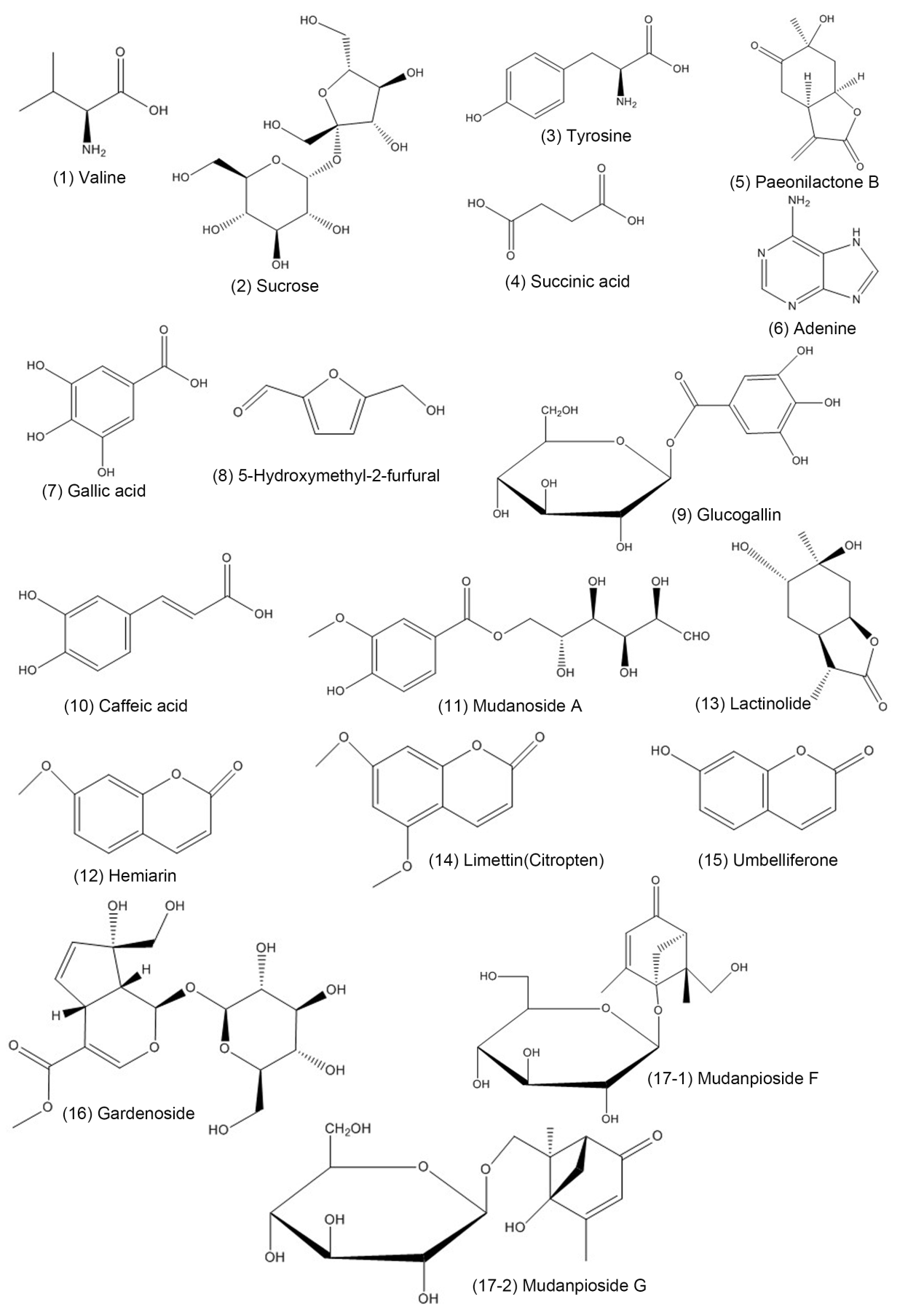


**Figure S3. The chemical structure of each component identified in JWXY capsule.**


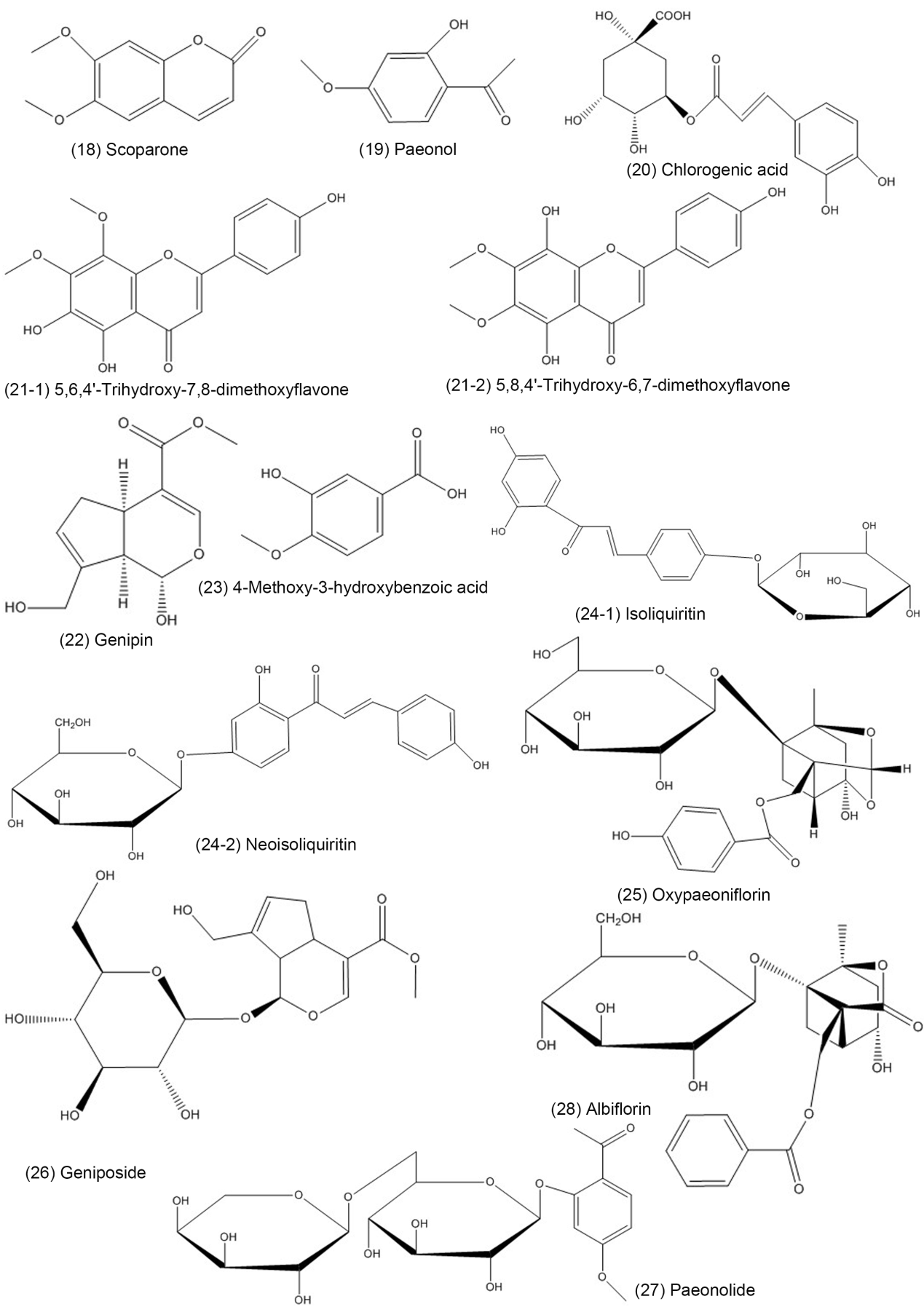


**Figure S4. The chemical structure of each component identified in JWXY capsule.**


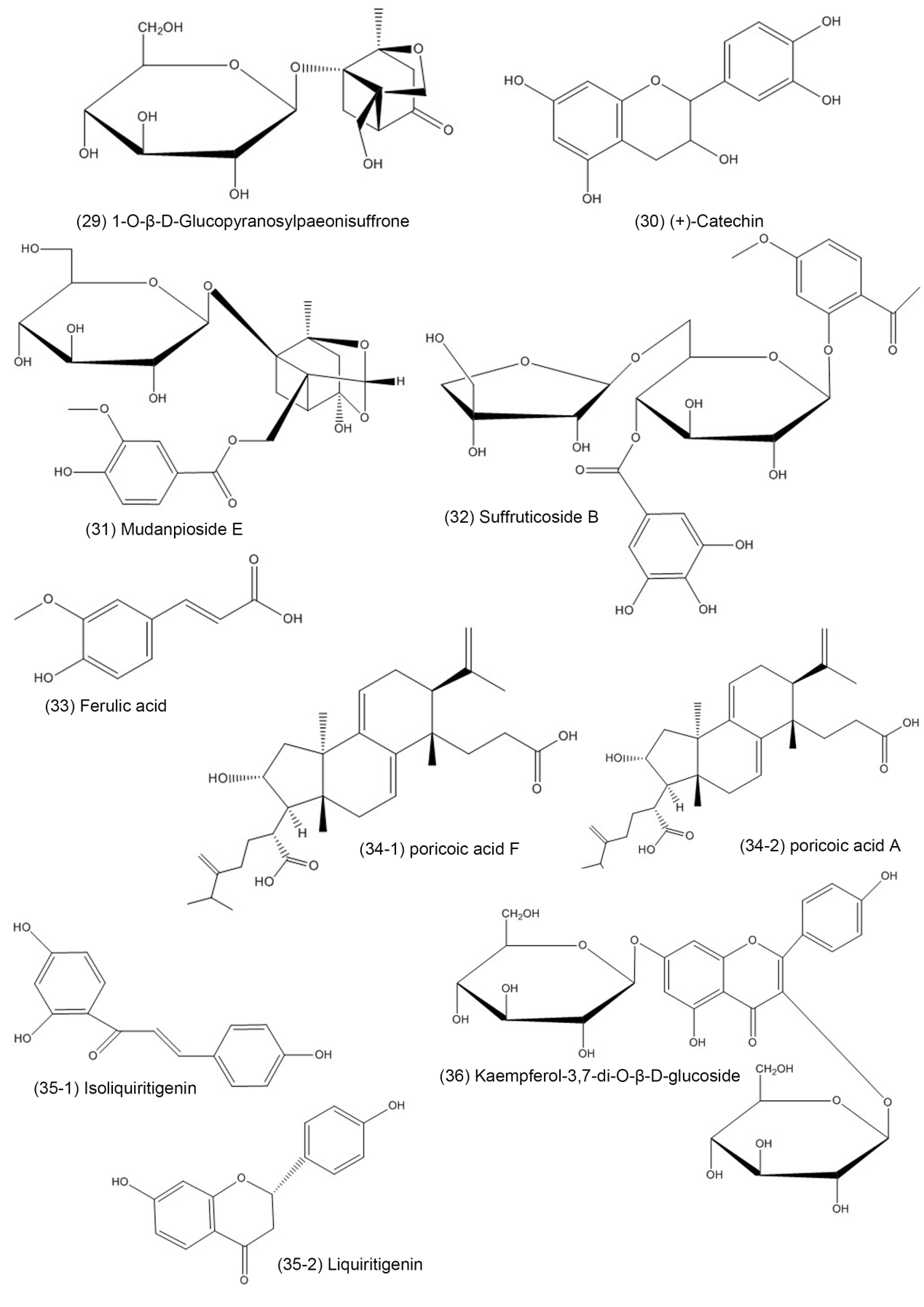
 **Figure S5. The chemical structure of each component identified in JWXY capsule.**


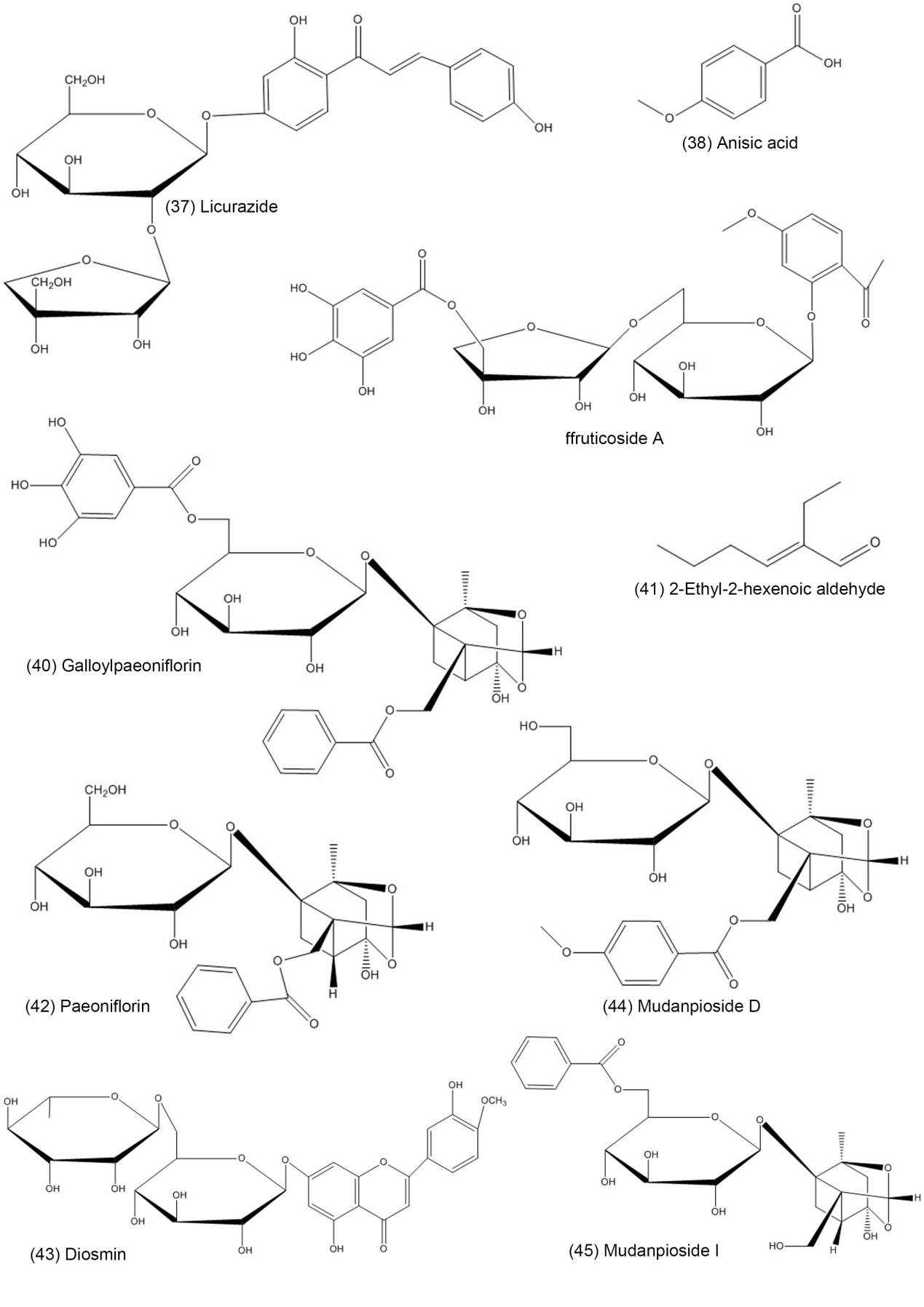
 **Figure S6. The chemical structure of each component identified in JWXY capsule.**


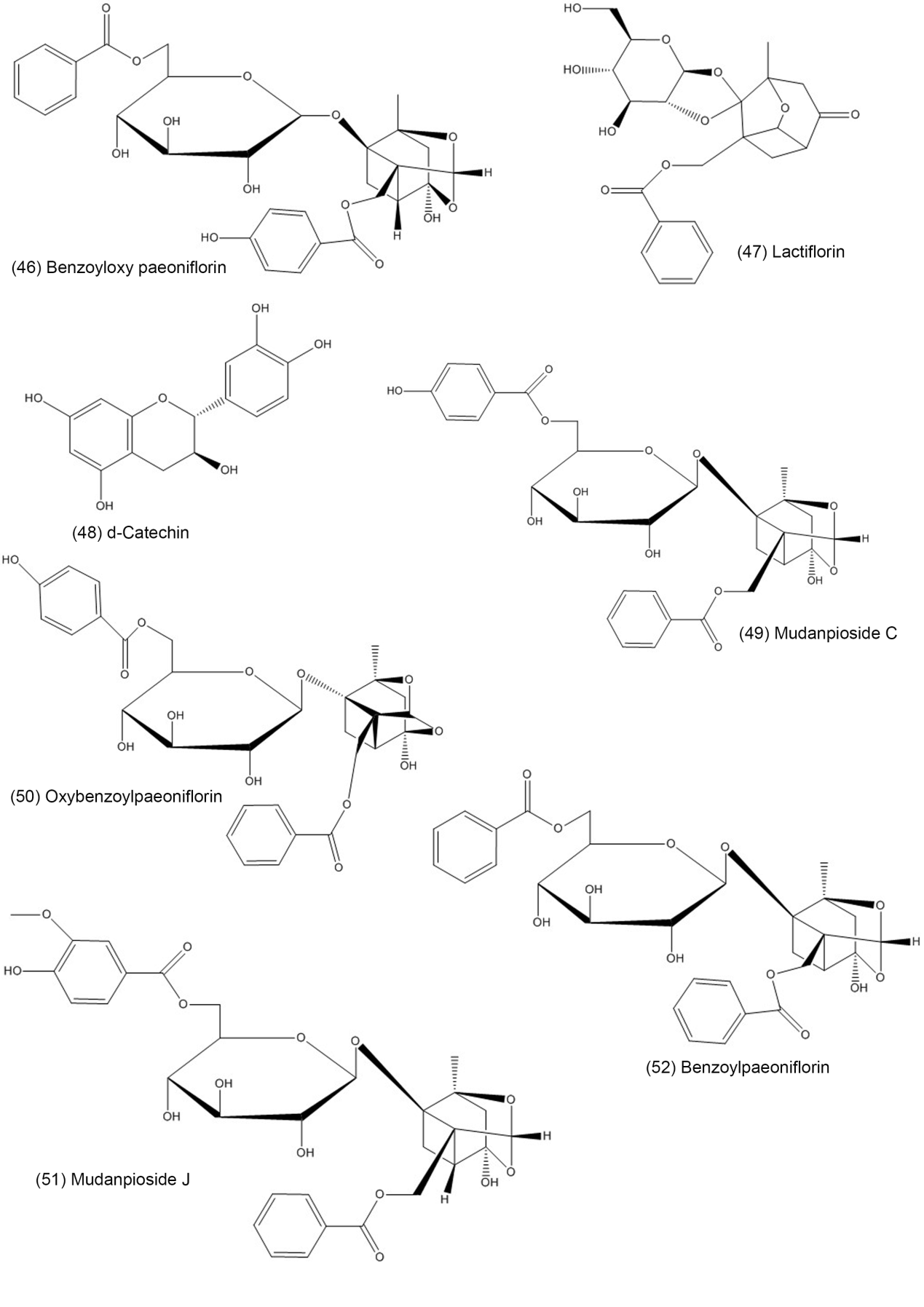
 **Figure S7. The chemical structure of each component identified in JWXY capsule.**


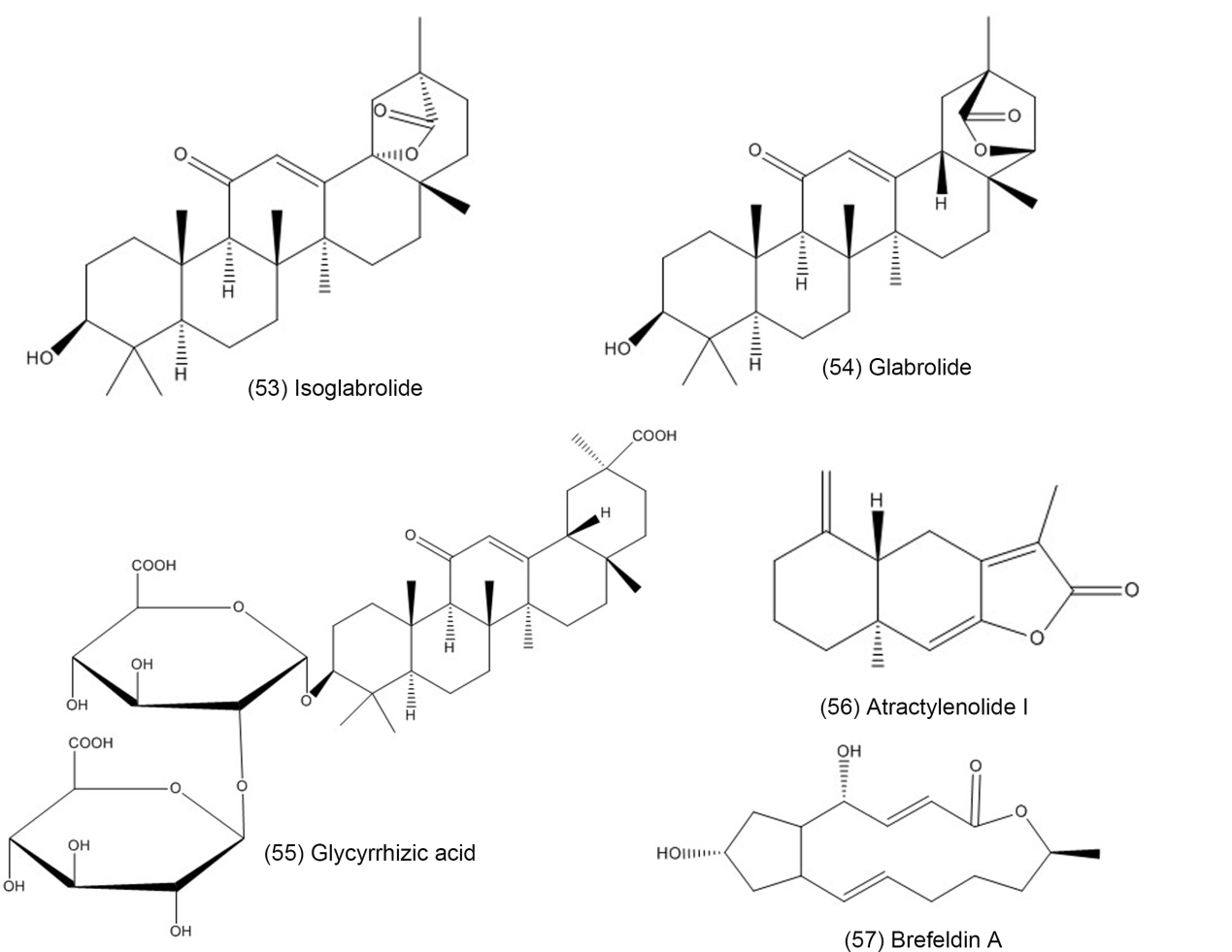
 **Figure S8. The chemical structure of each component identified in JWXY capsule.**


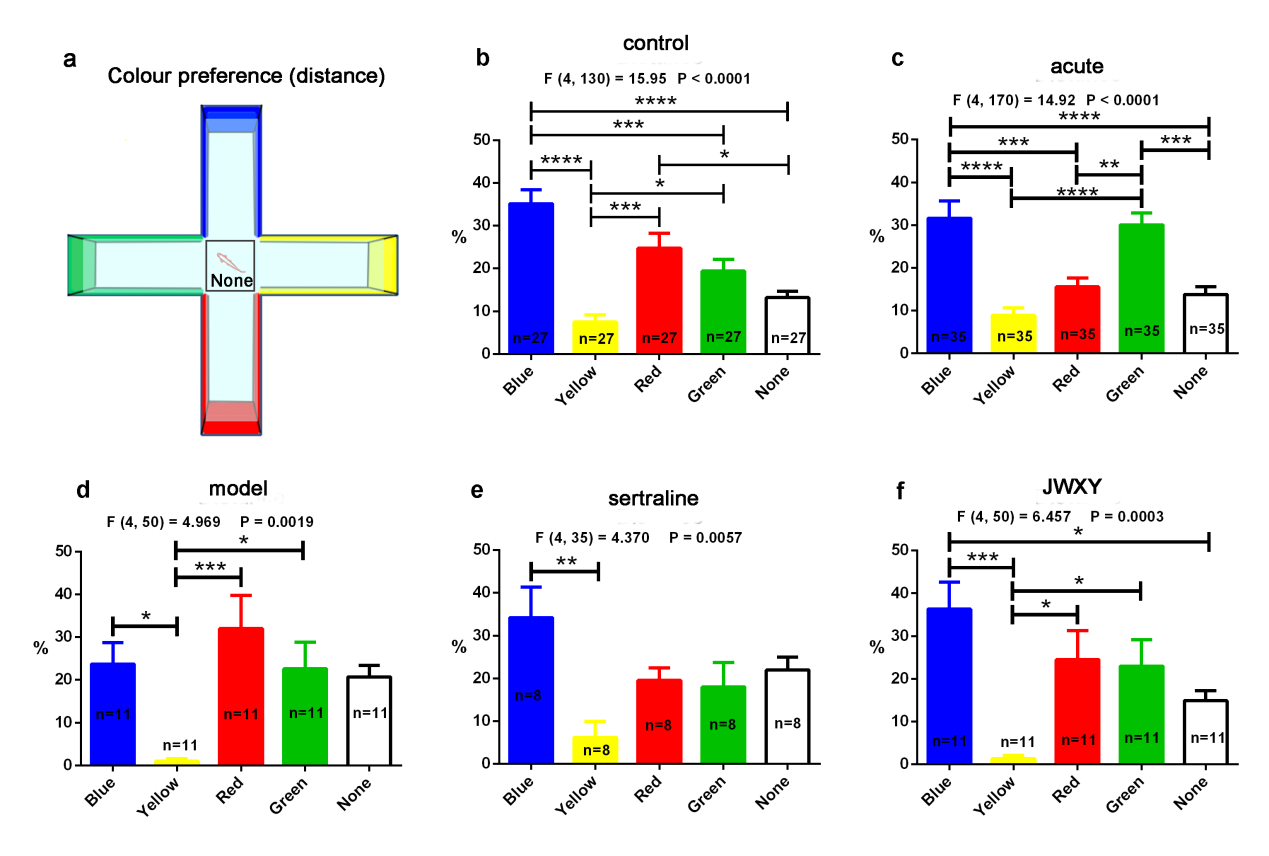


**Figure S9.** **Colour preference profiles of zebrafish exposed to sertraline and JWXY capsule after reserpine treatment in the remoulded offset** **cross maze test.** (a) Diagram of the remoulded offset cross maze and distribution of colours. The center of cross maze was denoted as None and zebrafish was started from here. (b) The distance travelled of control zebrafish in every colour arm. (c) The distance travelled of zebrafish treated with reserpine about 20 minutes (acute) in every colour arm. (d) The distance travelled of zebrafish exposed to system water after reserpine treatment (model) in every colour arm. (e) The distance travelled of zebrafish exposed to sertraline after reserpine treatment in every colour arm. (f) The distance travelled of zebrafish exposed to JWXY capsule after reserpine treatment in every colour arm. Control: untreated AB strain zebrafish. Acute: acute treatment with reserpine for 20 minutes. Model: after acute treatment with reserpine, zebrafish were exposed to system water for 7 days to generate the depression model. Sertraline: after acute treatment with reserpine, zebrafish were exposed to sertraline for 7 days. JWXY: after acute treatment with reserpine, zebrafish were exposed to JWXY capsule for 7 days. The data are expressed as the mean ± S.E.M. and were analysed by one-way ANOVA followed by the Tukey *post hoc* test. Significance was defined as **p* < 0.05, ***p* < 0.01, ****p* < 0.001 and *****p* < 0.0001.


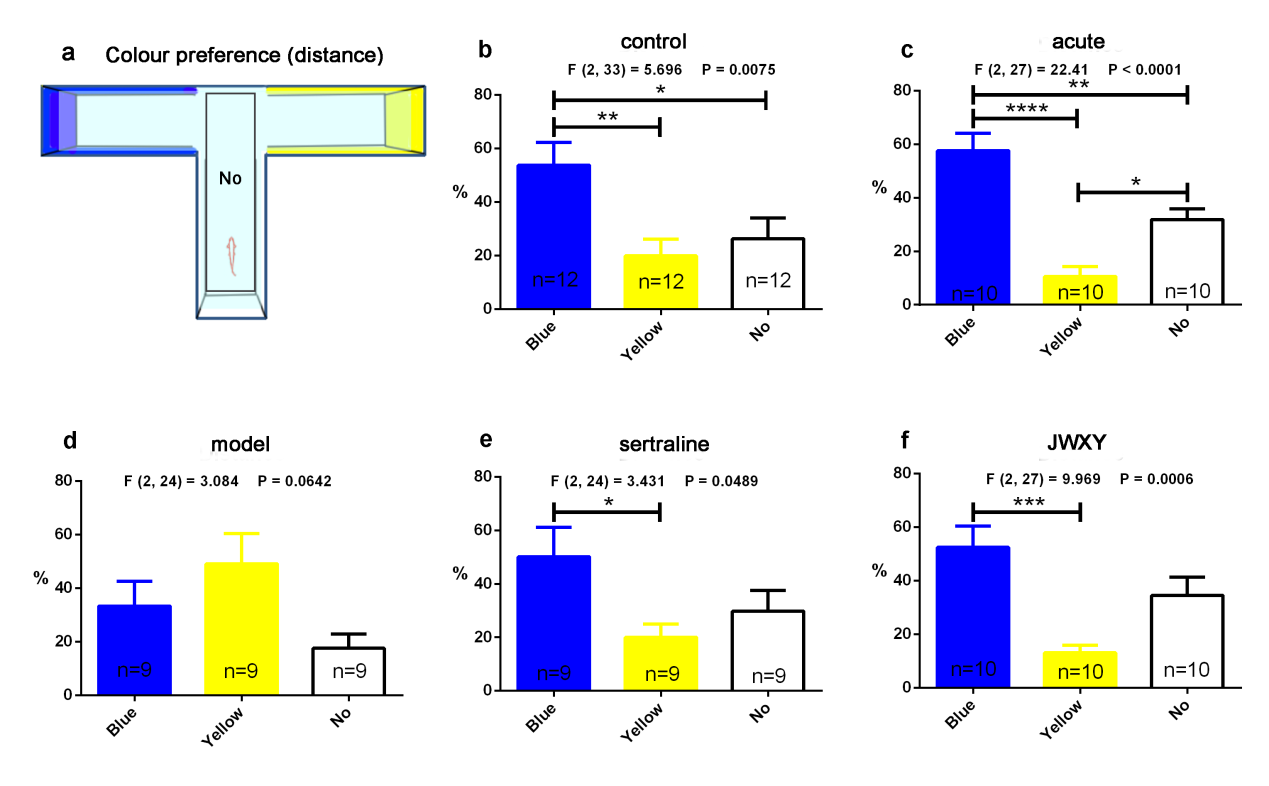


**Figure S10.** **Colour preference profiles of zebrafish exposed to sertraline and JWXY capsule after reserpine treatment in the T maze test.** (a) Diagram of the T maze and distribution of colours. The center of T maze was denoted as No and zebrafish was started from here. (b) The distance travelled of WT zebrafish in every colour arm. (c) The distance travelled of zebrafish treated with reserpine about 20 minutes (acute) in every colour arm. (d) The distance travelled of zebrafish exposed to system water (model) after reserpine treatment in every colour arm. (e) The distance travelled of zebrafish exposed to sertraline after reserpine treatment in every colour arm. (f) The distance travelled of zebrafish exposed to JWXY capsule after reserpine treatment in every colour arm. Control: untreated AB strain zebrafish. Acute: acute treatment with reserpine for 20 minutes. Model: after acute treatment with reserpine, zebrafish were exposed to system water for 7 days to generate the depression model. Sertraline: after acute treatment with reserpine, zebrafish were exposed to sertraline for 7 days. JWXY: after acute treatment with reserpine, zebrafish were exposed to JWXY capsule for 7 days. The data are expressed as the mean ± S.E.M. and were analysed by one-way ANOVA followed by the Tukey *post hoc* test. Significance was defined as **p* < 0.05, ***p* < 0.01, ****p* < 0.001 and *****p* < 0.0001.


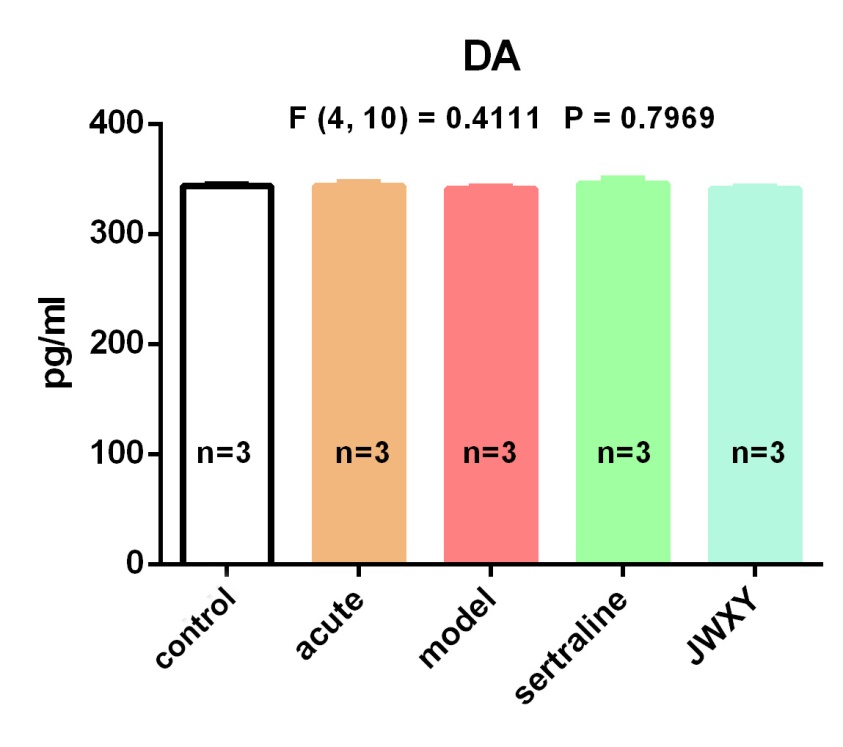


**Figure S11. The changes of sertraline and JWXY capsule treatment on dopamine (DA) of zebrafish.** Control: untreated AB strain zebrafish. Acute: acute treatment with reserpine for 20 minutes. Model: after acute treatment with reserpine, zebrafish were exposed to system water for 7 days to generate the depression model. Sertraline: after acute treatment with reserpine, zebrafish were exposed to sertraline for 7 days. JWXY: after acute treatment with reserpine, zebrafish were exposed to JWXY capsule for 7 days. The data are expressed as the mean ± S.E.M. and were analysed by one-way ANOVA followed by the Tukey *post hoc* test. Significance was defined as **p* < 0.05, ***p* < 0.01, ****p* < 0.001 and *****p* < 0.0001.
